# *Eucalyptus red grandis* pretreatment with protic ionic liquids: effect of severity and influence of sub/super-critical CO_2_ atmosphere on pretreatment performance†

**DOI:** 10.1039/d0ra02040k

**Published:** 2020-04-22

**Authors:** Francisco Malaret, Florence J. V. Gschwend, Joana M. Lopes, Wei-Chien Tu, Jason P. Hallett

**Affiliations:** Department of Chemical Engineering, Imperial College London London SW7 2AZ UK j.hallett@imperial.ac.uk http://www.imperial.ac.uk/people/j.hallett +44 (0)2075945388; High Pressure Process Group, Department of Chemical Engineering and Environmental Technology, University of Valladolid Spain

## Abstract

Deconstruction of lignocellulosic biomass with low-cost ionic liquids (ILs) has proven to be a promising technology that could be implemented in a biorefinery to obtain renewable materials, fuels and chemicals. This study investigates the pretreatment efficacy of the ionoSolv pretreatment of *Eucalyptus red grandis* using the low-cost ionic liquid triethylammonium hydrogen sulfate ([N_2220_][HSO_4_]) in the presence of 20 wt% water at 10% solids loading. The temperatures investigated were 120 °C and 150 °C. Also, the influence of performing the pretreatment under sub-critical and supercritical CO_2_ was investigated. The IL used is very effective in deconstructing eucalyptus, producing cellulose-rich pulps resulting in enzymatic saccharification yields of 86% for some pretreatment conditions. It has been found that under a CO_2_ atmosphere, the ionoSolv process is pressure independent. The good performance of this IL in the pretreatment of eucalyptus is promising for the development of a large-scale ionoSolv pretreatment processes.

## Abstract 2

Deconstruction of lignocellulosic biomass with low-cost ionic liquids (ILs) has proven to be a promising technology that could be implemented in a biorefinery to obtain renewable materials, fuels and chemicals.

## Graphical abstract

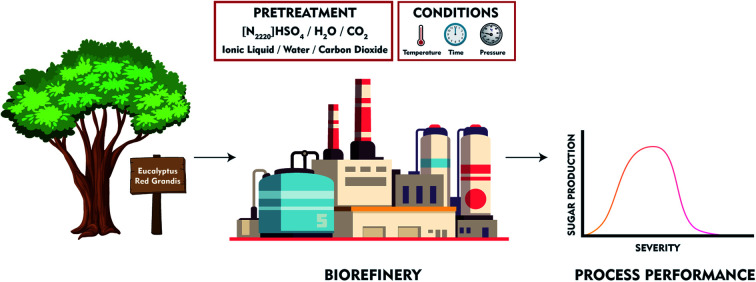

## Introduction

Lignocellulosic biomass is comprised of three biopolymers, namely cellulose, hemicellulose and lignin. Lignocellulosic-biomass derived energy and products are a promising alternative to petroleum derived fuels and chemicals as a way of reducing CO_2_ emissions of the energy, chemical and fuel sectors. In order to achieve this, it is necessary to first deconstruct and/or fractionate biomass though a process known as pretreatment.^1^ Different pretreatment methods, such as steam explosion,^2^ AFEX,^3^ concentrated acid,^4^ dilute acid,^5^ hot water,^6^ organosolv^7,8^ and ionic liquid (IL) pretreatments,^9–11^ including the ionoSolv process,^12–15^ affect lignin content and structure, and cellulose crystallinity, accessibility and hydrolysis kinetics in different ways. Biomass fraction using protic ionic liquids have been discussed in the scientific literature.^16,17^

The pretreatment of biomass with ionic liquids has been extensively studied using 1-ethyl-3-methylimidazolium acetate [C_2_C_1_im][Ac] with various feedstocks, *e.g.* switchgrass,^18^ wood flour,^19^ corn stover,^20^ bagasse,^21^ rice straw,^22^ cotton linters and eucalyptus.^23^ This IL presents low toxicity and viscosity, but despite promising results there are some limiting factors for industrial application, such as high IL cost and stability^24^ and the requirement for very low water content for optimum performance.

A comparative study on low-cost ILs suggests that ILs obtained through an improved design based on the hydrogen sulfate anion are cost-effective delignifiers.^25^ Alkylammonium hydrogen sulfate ILs can be produced at much reduced cost compared to alkylimidazolium ILs due to cheaper starting materials and ease of synthesis, providing much of the efficacy compared to [C_2_C_1_im][Ac] at a fraction of the cost.^25^ The utilization of these types of IL–water mixtures to extract the lignin and hemicellulose while leaving behind a cellulose-rich pulp is known as the ionoSolv process.^26^ This process has been used to efficiently pretreat the grass *Miscanthus giganteus*,^15^ sugarcane bagasse^27^ and willow^28^ using triethylammonium hydrogen sulfate, [N_2220_][HSO_4_], while a similar IL, *N*,*N*-dimethylbutylammonium hydrogen sulfate [N_4110_][HSO_4_], has been used to effectively pretreat the softwood pine^14^ and other feedstocks.^29^ Protic IL pretreatment is able to remove more lignin than dilute acid pretreatment, reduce the total process time to produce high yields of sugar from the recovered product and produce less degradation products.^18^ It also improves the enzymatic hydrolysis efficiency as a results of the disruption of the biomass structure, leading to lower enzyme requirement and shorter hydrolysis time.^30^ Although some ILs are capable of dissolving or swelling cellulose, the presence of water in these protic IL systems retards the transformation of cellulose I to amorphous/cellulose II while amorphous bits of the cellulose and virtually all of the hemicellulose are removed, thus the overall Crystallinity Index (CrI) of the pulp increases with water content in the IL.^31^

In addition to performance, ionic liquid recycling is a key element for process economics. In a study performed on eucalyptus pretreated with 1-allyl-3-methylimidazolium chloride [amim][Cl] and 1-butyl-3-methylimidazolium acetate [C_4_C_1_im][Ac], the glucose yield dramatically decreased when recycled IL was used, dropping from 76.3 to 54.3% for [amim][Cl] and from 88.9% to 72.8% for [C_4_C_1_im][Ac], after 4 cycles.^32^ IL degradation was also observed. This decrease in performance as IL is recycled will result in prohibitive processing cost as the IL inventory needs to be replaced on a continuous basis or regenerated *via* more stringent purification systems to keep biomass deconstruction performance. In the case of hydrogen sulfate based ILs, it has been shown that the IL can be easily recovered and re-used without loss in performance. In fact, rather than a decrease in performance, a slight increase of the saccharification yields after 4 cycles, from 79.1% to 81.1% for *Miscanthus* pretreatment using [N_2220_][HSO_4_] with 20 wt% water has been reported.^33^

Dealing with highly viscous fluids in industrial installations is not desired as this will increase the pumping costs and reduce the efficiency of mass transfer controlled processes. For these reasons, the addition of molecular solvents to an IL has been used to decreases the solution viscosity.^34^ From a process perspective, using aqueous IL solutions as a pretreatment medium is more favoured over the use of anhydrous ILs. The presence of water can potentially reduce the amount of washing solvent, reduce viscosity and significantly reduce energy and costs associated with IL recycling. As an alternative to water or organic solvents, *e.g.* alcohols,^35,36^ the addition of pressurized carbon dioxide has also been used to decrease the viscosity and melting point of high melting point ILs capable of dissolving cellulose.^37,38^ CO_2_ as a co-solvent has the advantages of being non-toxic, cheap, and easily separable from the IL by depressurization. Thus, supercritical CO_2_-IL systems have much potential as biphasic systems for reactions and separations^39^ but so far have never been studied for biomass pretreatment using protic ILs.

CO_2_ (without ILs) has been used in lignocellulose pretreatment in the form of CO_2_ explosion pretreatment, typically requiring high pressure, between 20 and 27.6 MPa (200 and 276 bar).^40^ A comparison between supercritical CO_2_ and conventional acid pretreatments showed similar effects on cellulose crystallinity and improvement of the enzymatic digestibility for these two methods, however the supercritical CO_2_ pretreatment presents lower operating costs and better environmental performance than conventional acid pretreatment.^41^ A combination of CO_2_ and IL pretreatment thus appears to be a promising candidate for industrial application, on account of cost-effectiveness of ILs and the potential of the addition of CO_2_ to reduce the viscosity of the reaction mixture.

Eucalyptus has been proposed as a promising raw material for biorefining as large quantities of residues are available from fast grown plantations. Eucalyptus has been used for fuel, charcoal, pulp and paper and widely employed for sawn timber production.^42^ The rapid growth and their ease to adapt to various environmental conditions makes this species a potentially abundant source of fermentable sugars. A high-level assessment of 2G ethanol production, with several feedstocks *via* diluted acid pretreatment, showed that eucalyptus yielded the lowest ethanol production cost when compared to poplar, straw and switchgrass.^43^ IL pretreatment of eucalyptus has been explored with hydrogen sulfate based IL^44^ and also with higher cost ILs *e.g. Eucalyptus globulus* pretreatment with [C_2_C_1_im][Ac]^45^ and [C_2_C_1_im][Cl],^46^ and *Eucalyptus grandis* pretreatment with pyrrolidinium acetate [Pyrr][Ac] and [C_4_C_1_im][Ac].^47^

The aim of the current study is to achieve high saccharification yields from *Eucalyptus red grandis* pretreatment with the inexpensive protic IL triethlyammonium hydrogen sulfate with minimum severity (time and temperature). Additionally, we investigate the effect of pressurized CO_2_ on the deconstruction during ionoSolv pretreatment. Both the cellulose rich pulp and the lignin fraction are analysed and predictive models for the lignin, cellulose and hemicellulose extraction proposed.

## Results and discussion

### Pretreatment experimental setup, temperature profiles and severity factors

The bench-scale pretreatment experiments were carried out according to a published procedure^48^ in unstirred 15 mL glass pressure tubes from ACEglass. They were loaded with ground biomass (air-dried) and solvent ([N_2220_][HSO_4_] containing 20 wt% water) in a 1 : 10 g/g biomass (on an oven dried basis) to solvent ratio at room temperature. The samples were then placed inside a fan-assisted oven preheated to a fixed temperature for the duration of the pretreatment and finally taken out of the oven and allowed to cool down by natural convention.

The temperature profiles of the reaction medium were measured with the help of a FEP (fluorinated ethylene propylene) coated K thermocouple (TC direct) placed in a glass pressure tube containing ionic liquid solution. The temperature profiles for the experiments are given in Fig. S1 (ESI†). As shown in previous work^15^ the reaction takes place in a non-isothermal regime, and the system reaches ∼99% of the target temperature after 30 minutes (independent of the reaction temperature). The cooling time from reaction temperature to room temperature is also ∼30 minutes.

A set of larger scale experiments (eucalyptus mass increase of ∼45%, from ∼1.1–1.6 gr air dried) was conducted in 100 mL glass pressure tubes from ACEglass at 180 °C, keeping the same biomass to solvent ratio of 1 : 10 g/g (on an oven dried basis). Not only the heating profile inside the large pressure tubes was impacted (increased mass and reduced contact of the medium with the wall of the container) but the increased container volume caused an increase in its headspace leading to excessive water evaporation from the ionic liquid, resulting in very poor performance. The temperature profiles are given in the ESI† to make researchers aware how experimental modifications (mass increased by 45% and container geometry change, form 15 mL to 100 mL) can have significant impact on the outcome of pretreatment experiments, specially at short times. This is mentioned in this work only to illustrate the complexities of process scale-up and will not be further discussed here.

The severity factor relationship has been calculated according to the method developed by Chornet and Overend, which takes into account the pretreatment temperature and time to predict conditions that result in similar yields from the breakdown of hemicellulose to its component sugars according to the following relationship:^49,50^1
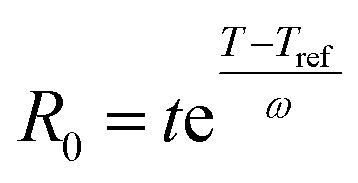
where *R*_0_ is the severity factor (assuming here a constant acidity), *T* is the temperature of the reaction medium in °C, *T*_ref_ is a reference temperature in °C, *t* is the time in minutes and *ω* a parameter expressing the effects of temperature in the specific reaction considered.

Typical values used to evaluate the severity factor during biomass fractionation are 100 °C as reference temperature and 14.75 for *ω*.^51^ An *ω* value of 14.75 means that the rate of reaction doubles ten degrees above the reference temperature, all the other variables remaining constant. In other words, the exponential term is consistent with the heuristic that the reaction rate will double for every 10 °C increase in temperature. Eqn (1) predicts that the value of *R*_0_ remains the same if the time *t* is divided in half for every 10 °C increase in temperature. It is noteworthy to mention that the typical values for *T*_ref_ and *ω* used in this work are the same, regardless of the biopolymer, to allow comparisons with other pretreatment methods, which also used those values. Technically speaking, the *ω* value is related to a pseudo-activation energy of the process being considered and the biomass, and should be evaluated for each biomass fraction.^51–53^ For reference, the activation energy of cellulose hydrolysis ranges in 87.73–179 kJ mol^−1^,^52,53^ hemicellulose fraction 129 kJ mol^−1^^54^ and for delignification 89–131 kJ mol^−1^.^51^

As the temperature profiles are not isothermal, the severity factors have been calculated by integration of the temperature profiles over time, according to:2
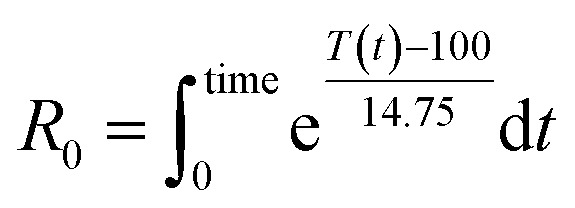


The integral form of the equation has some similarities to the H-factor equation that was developed by Rydholm to relate time and temperature for delignification in Kraft pulping:^49^3
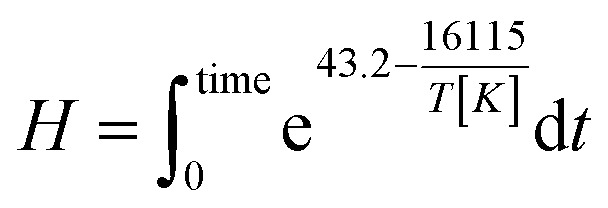


The numerical results of the calculated severity factors and H-factors are given in Table S1 (ESI†). As expected, the errors of assuming isothermal conditions are greater at short pretreatment times.

### Effect of intensified [N_2220_][HSO_4_] on pretreatment

**Fig. 1:**
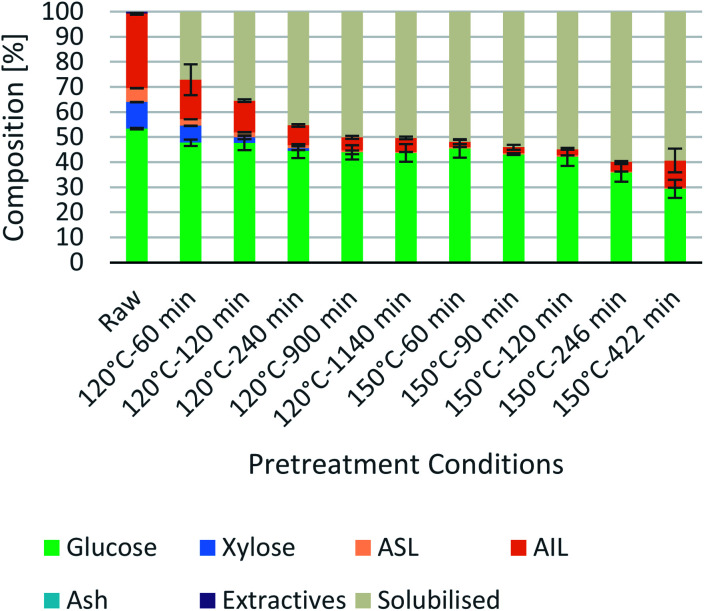
Eucalyptus pulp composition at different pretreatment conditions using a 1 : 10 g/g biomass to solvent ratio in 80% [N_2220_][HSO_4_] with 20 wt% water.

**Fig. 2:**
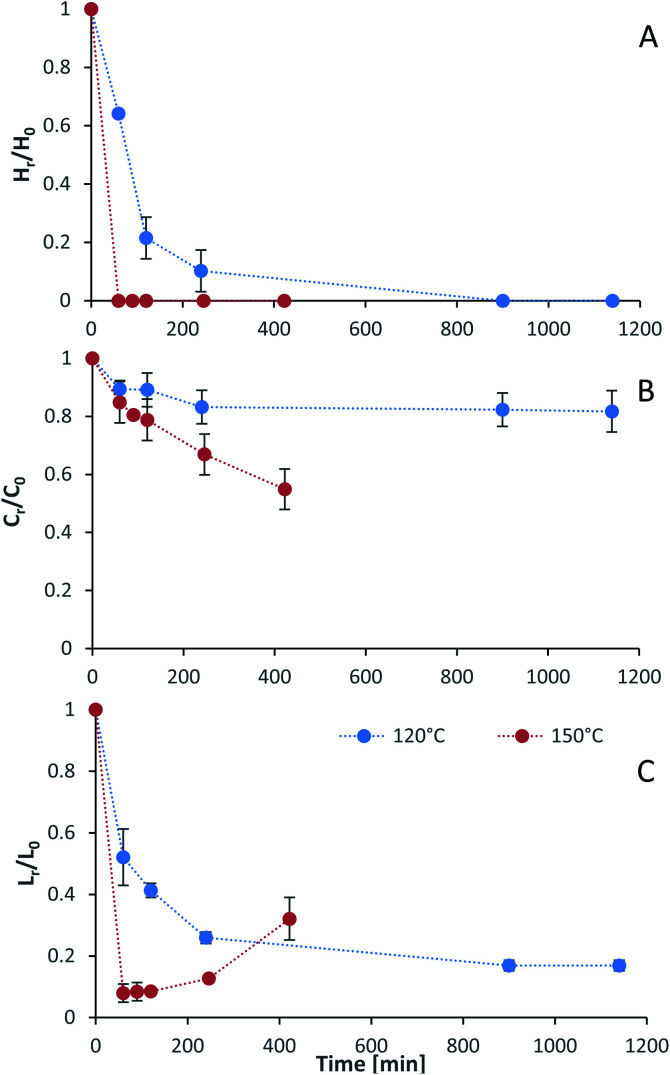
The mass fraction of (A) hemicellulose [*H*_r_/*H*_0_], (B) cellulose [*C*_r_/*C*_0_], and (C) lignin [*L*_r_/*L*_0_] remaining in the pulp after pretreatment as a function of time at two temperatures (blue line 120 °C, red line 150 °C). Dotted lines were added to guide the reader's eye and are not a kinetic fit. Eucalyptus was pretreated at a 1 : 10 g/g biomass to solvent ratio in 80% [N_2220_][HSO_4_] with 20 wt% water.

Process intensification affects pulp composition in different ways. Pulp yield and composition as a function of pretreatment conditions are given in Fig. 1. As expected, the pulp yields decrease with increasing severities (temperature and/or pretreatment time). Fig. 2 shows the amount of material remaining in the pulp after pretreatment relative to the initial amount as a function of time for each main biomass component (mass fraction of hemicellulose [*H*_r_/*H*_0_], cellulose [*C*_r_/*C*_0_], and lignin [*L*_r_/*L*_0_]).

As can be seen in Fig. 2(A), the hemicellulosic fraction [*H*_r_/*H*_0_] was quickly removed from the pulp and complete removal can be achieved at high severity factors. Comparing with previous work it appears that the hemicellulosic fraction from eucalyptus can be removed more easily than the one from willow^28^ or *Miscanthus*.^15^ Complete hemicellulose removal was achieved at 120 °C and pretreatment times equal to or longer than 240 min, and for 150 °C at pretreatment times longer than 60 min.

The IL can also degrade the glucan fraction (lower *C*_r_/*C*_0_, Fig. 2(B)), which is more evident at higher temperatures (150 °C). This is consistent with findings for other hardwoods, such as willow, in which the glucan fraction was also degraded upon high-temperature IL pretreatment.^28^ It has been noticed that the glucan fraction in these hardwoods decreases more quickly when compared to *Miscanthus*.^15^ There are several reasons that might explain this, for example differences in the cellulose nature (crystallinity) between those biomasses. The analytical method does not discriminate between glucose monomers coming from cellulose or hemicelluloses, therefore, the hardwoods might contain glucose originating from the hemicellulosic fraction but are not detected as such. Another reason might be that the IL was slightly acidic due to errors in the synthesis method. In previous work it was shown that the acid/base ratio of the IL affects the saccharification yields and the amount of glucan remaining in the pretreated solids, where an excess of acid leds to a more significant degradation of the glucan fraction.^28^

Residual lignin in the pulp (*L*_r_/*L*_0_, Fig. 2(C)) decreases with the severity and then increases at high severities. This is consistent with previous work with *Miscanthus*,^15^ bagasse,^27^ pine^14^ and willow^28^ and can be explained by precipitation of condensed lignin and pseudo-lignins back onto the pulp surface.

At 120 °C the lignin content decreased monotonically, and it did not show an increase even after long pretreatment times (1140 min or 19 h). At 150 °C, the lignin content rapidly decreases at short pretreatment times and then gradually increases, reaching a minimum lignin content of ∼3% in the pulps at short pretreatments (∼8% of residual lignin [*L*_r_/*L*_0_]).

**Fig. 3:**
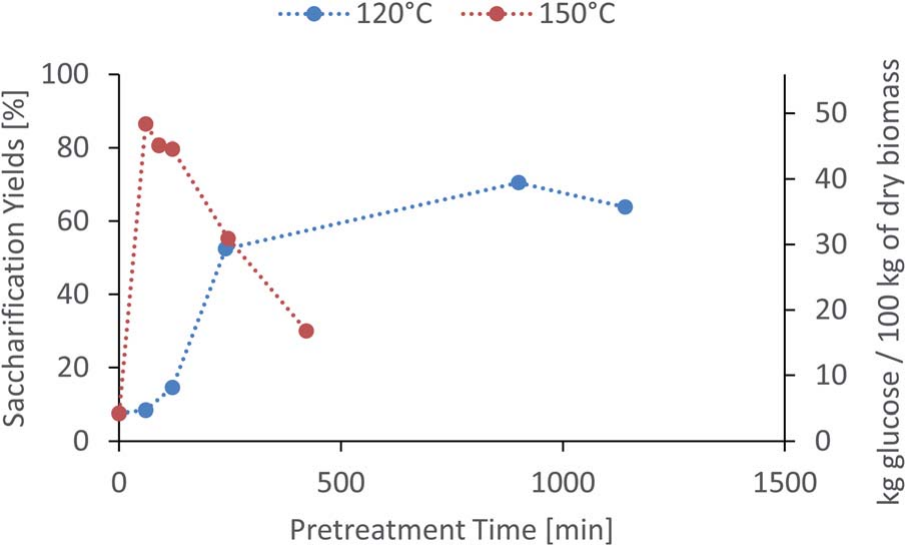
Glucose yield of pretreated eucalyptus at a 1 : 10 g/g biomass to solvent ratio in 80% [N_2220_][HSO_4_] with 20 wt% water after enzymatic saccharification.

The saccharification yield follows a similar trend; it increases with increased severity, reaching a maximum, then decreasing at very high severities (Fig. 3). A plot of saccharification yields *vs.* delignification (Fig. S5 in ESI†) shows some degree of correlation as previously shown for softwoods.^14^ The saccharification yields peaked at the minimum lignin content and reached a value of 86 ± 1% (48.5 ± 0.5 kg glucose/100 kg dry biomass) (150 °C for 60 min), which is comparable to other pretreatment methods, for example 84 ± 2% glucose yield after enzymatic saccharification of dilute acid pretreated eucalyptus chips,^55^*Eucalyptus globulus* pretreatment with [C_2_C_1_im][Ac]^45^ yielded to 85–95% glucose yield recovery depending on the eucalyptus mutant used, and the use of [C_2_C_1_im]Cl on *Eucalyptus globulus* Labill. resulted in a maximum glucose yield of 82.2% wt.^46^

In summary, the compositional analysis and saccharification data show that increasing the temperature from 120 °C to 150 °C can lead to pulps with a high cellulose content, no hemicelluloses and small quantities of lignin and with high enzymatic digestibility; all of these with a reduction in the pretreatment time, which may lead to significant savings in the capital cost of the pretreatment reactor. Optimisation of pretreatment conditions will be required to prevent excess overtreatment and maintain a high pulp quality.

A work on delignification of *Eucalyptus nitens* using [C_4_C_1_im][HSO_4_] and [N_2220_][HSO_4_] was recently published.^44^ In this work, eucalyptus was first hydrolysed in water at 195 °C (autohydrolysis) to remove the hemicellulosic fraction and water soluble components (pulp yield 72.8% and 80.7% xylan solubilisation). The subsequent delignification with [N_2220_][HSO_4_] removed the lignin and yielded a pulp with a residual xylan content ranging from 3.3–3.8% and lignin content ranging from 18.2–13.2, depending on the severity. Compared to the *Eucalyptus red grandis* results, a xylan fraction was present in the pulps for all experimental conditions. Even though it seems that the hemicellulosic fraction in *Eucalyptus red grandis* is easier to remove than in *Eucalyptus nitens*, more experiments are required to conclude this point. The reason for this is that the pretreatments were not carried out under the same conditions and different wood sources should be tested.

It has been reported that during autohydrolysis, polysaccharide hydrolysis evolves uniformly due to the presence of acetic acid catalyst which is formed due to the cleavage of acetyl groups present in the hemicellulosic fraction of some lignocellulosic biomass.^44^ It has been shown that the hydrogen sulfate-based ionic liquids perform extremely well, even in the absence of the autohydrolysis step. However, the autohydrolysis step, or equivalent method, might be used in large scale processes to remove the hemicellulosic fraction that will solubilise in the ionic liquid, and degrade during the pretreatment conditions, yielding pseudo-lignins and other degradation products.^56^

From these studies, it is hard to conclude which eucalyptus specie will be more suitable for biofuel production as enzymatic digestibility of *Eucalyptus nitens* pulps were not reported.^44^ However, in terms of pulp composition, both species led to high-cellulose content pulps.

It has been noticed that most papers report glucan recovery as a percentage of the maximum theoretical. This relative metric is useful for comparing trends during biomass fractionation but of little utility to evaluate the potential of a given feedstock for biomass production, due to differences in biomass composition. Therefore, the results here have been expressed in kg of glucan per 100 kg of biomass and compared with *Eucalyptus nitens* (Fig. S9 in ESI†).^44^ The trends are as expected, the glucan fraction decreases with increasing pretreatment times. The sample of *Eucalyptus red grandis* used in this study had a high initial glucan content when compared to literature values 39.9–49%.^57,58^ To draw a conclusion about the best eucalyptus species to use as feedstock for a given purpose, several samples should be analysed in order to have a statistically significant amount that will allow to assess the variability between trees of different ages, grown in different locations, *etc.* In this study it has been assumed that the chemical composition of the sieved samples (180–850 μm, 20 + 80 US mesh scale) is the same between the different fractions recovered and the chemical composition of the plank used is representative of all the zones of the tree.

### Predictive models

For accurate pretreatment performance predictions, detailed kinetic models need to be developed for each biomass component. As mentioned in our previous work,^15^ determining the kinetic parameters of heterogeneous reactions of biomass solubilization for non-isothermal systems is complex and therefore has been excluded from the scope of this work.

**Fig. 4:**
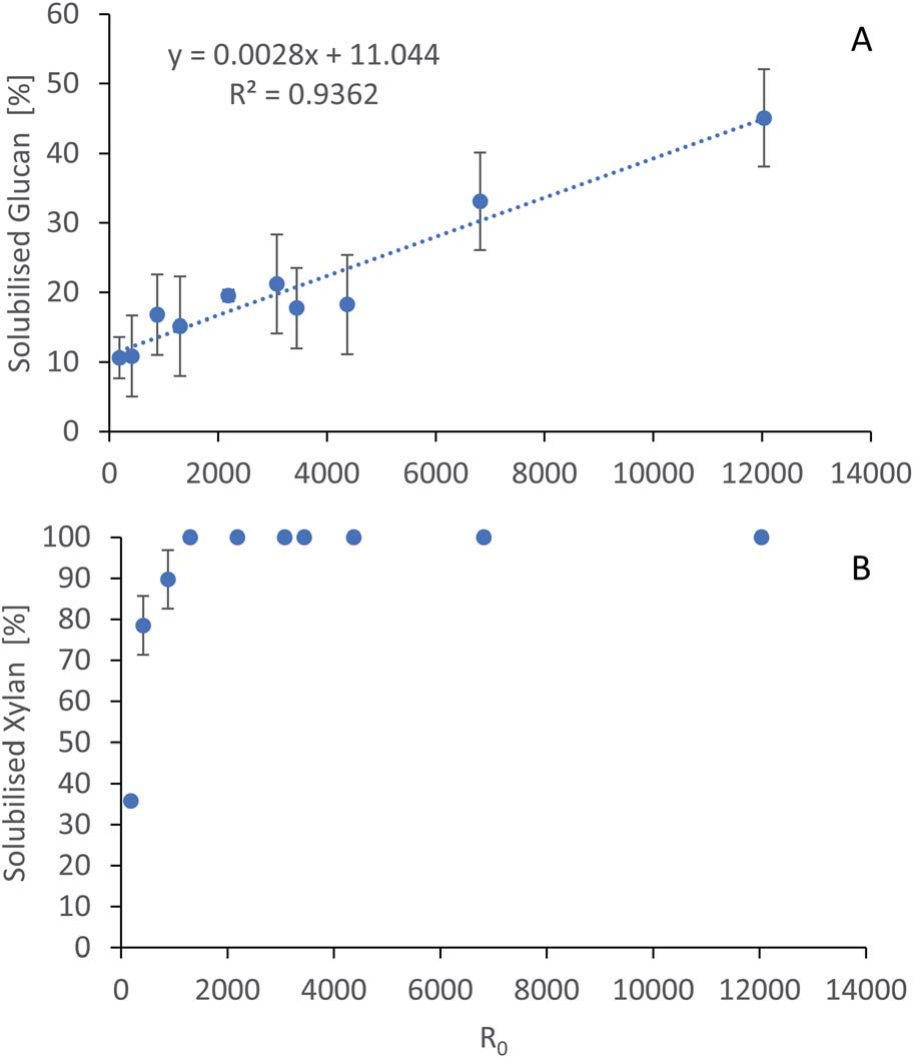
Polysaccharide dissolution as a function of the severity factors. (A) Glucan. (B) Xylan. Eucalyptus was pretreated at a 1 : 10 g/g biomass to solvent ratio in 80% [N_2220_][HSO_4_] with 20% water.

**Fig. 5:**
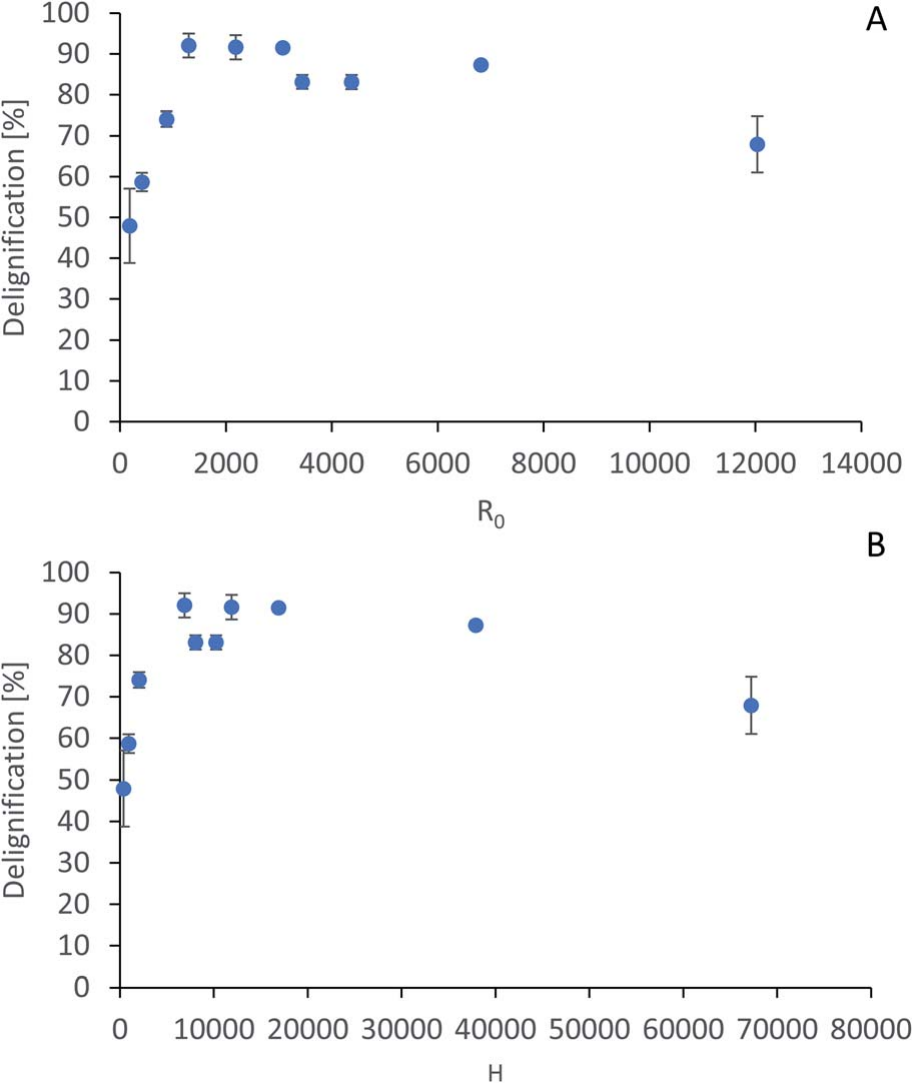
Predictive models for lignin removal. (A) Lignin removal as a function of severity factor. (B) Lignin removal as a function of H-factors.

The severity factors can be used as a simplified approach to predict the simultaneous effects of temperature and time on the pretreatment performance, as no reaction mechanistic details nor parametric fittings are required. To account for the non-isothermal nature of the experiments, the severity factors have been calculated with eqn (2) and (3). Fig. 4 shows the results of glucan and hemicellulose removal from the pulp as a function of the calculated severity factors according to eqn (2). From Fig. 4(A) it can be seen that glucan solubilization follows a linear trend within the range of severity factors spanned. This line does not go through the origin, suggesting that there must be a glucan fraction within the biomass that is easier to hydrolyse, and may be contained within the hemicellulose, as previously mentioned. Regarding the hemicellulose removal, it increases at low severity and it is complete at severities of ∼1300 (Fig. 4(B)). The lignin removal as a function of the severity factor (eqn (2)) and H-factors (eqn (3)), are shown in Fig. 5. Delignification shows maximum values in the range of *R*_0_ 1300–6900 or H 6900–16 900 (Fig. 5). Below this range, the lignin removal is insufficient and above, the condensation reactions and pseudo-lignin formation lead to a decrease in the delignification as previously explained. It seems that the pretreatment performance can be correlated with the severity factors and H-factors, which shows promise as a predictive tool for process design.

### Influence of pressure and CO_2_ atmospheres on pretreatment

It has been shown that there is a significant influence of the atmosphere composition on the liquefaction and depolymerization of wood in the IL 1-ethyl-3-methylimidazolium chloride [C_2_C_1_im]Cl.^59^ To address the influence of CO_2_ atmospheres on the performance of the pretreatment, a pressurised Parr reactor with a temperature controller was used. To prevent sample contamination, the Parr reactor internals (thermocouple well) was coated with PTFE and the pretreatment was carried out in a glass liner fitted to the rector. The same solvent and solid loading were used as for the bench-scale experiments under normal atmosphere. CO_2_ was loaded into the reactor at room temperature without purging the air inside. Two sets of experiments were performed: low severity (xL), with a final pressure of 2.1 ± 0.2 MPa (20 ± 2 barg), and high severity (xH) 8.3 ± 0.2 MPa (82 ± 2 barg) and a control without CO_2_ (x0). For each atmosphere, two pretreatment times were tested: short time (30 min), *i.e.* low severity (Lx), and long pretreatment time (4 hours), *i.e.* high severity (Hx). The experimental conditions are summarized in Table S2 (ESI†) and the temperature profiles are shown in Fig. S4 (ESI†).

**Fig. 6:**
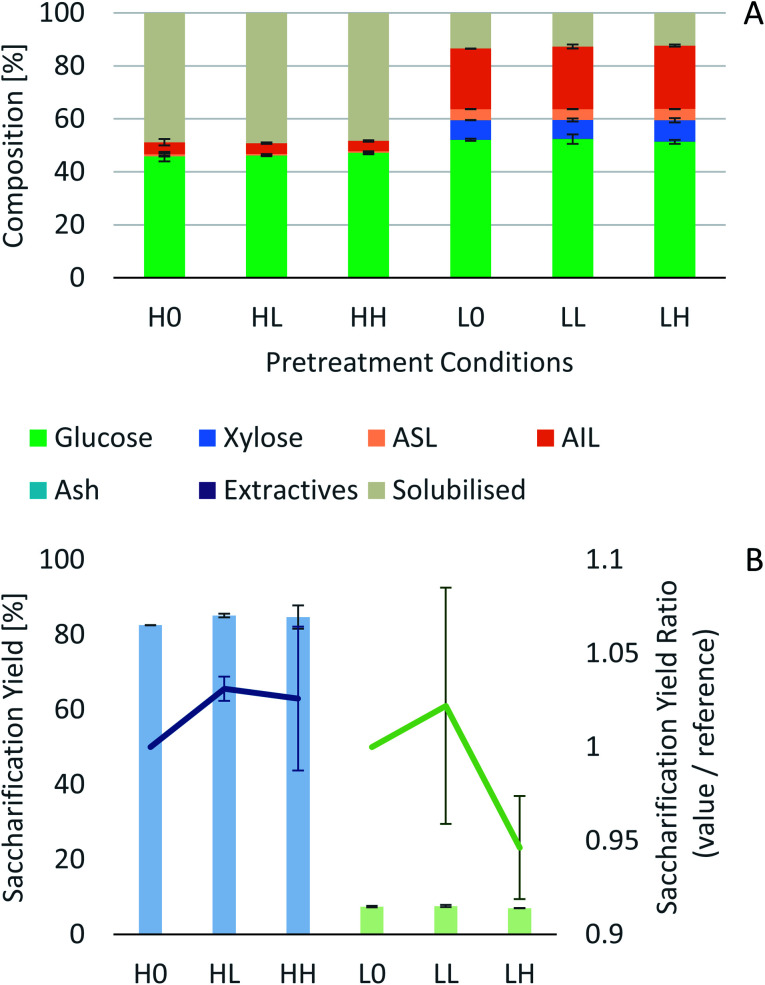
Effect of CO_2_ atmospheres on eucalyptus pretreatment. (A) Pretreated pulp compositions. (B) Saccharification yield of pretreated pulps (bars – left axis) and normalized saccharification yields relative to the control experiments (lines – right axis).

The experimental results of the pretreatment performed under CO_2_ atmosphere are shown in Fig. 6. Overall, there are no significant changes between performing the IL pretreatment under CO_2_ atmospheres regardless of whether the internal CO_2_ pressure is below or above the critical point (304.25 K or 31.10 °C and 7.384 MPa (73.84 bar)^40,60^), as can be seen in Fig. 6. The saccharification yields of the experiments show that there are no significant changes in the pretreatment performance with the addition of CO_2_. There is a slight increase (<2%) in the saccharification yields at 2.1 MPa (20 barg) of CO_2_ and no significant changes at 8.3 MPa (82 barg). An ANOVA: single factor analysis performed in excel (ESI†) shows that the differences between each experimental set are not statistically significant (*F* < *F*_cri_) with 95% certainty (alpha = 0.05). Therefore, it can be concluded that the pretreatment performance is unaffected by CO_2_ pressure and likely unaffected by other inert gases such as N_2_. It remains unclear if O_2_ will have a significant effect on pretreatment performance, as this gas showed an important influence in the biomass fractionation with the IL [C_2_C_1_im]Cl.^59^ However, the use of O_2_ may create additional safety issues, and will not be explored. Better options to boost sugar recovery would be to optimize the severity factors (pretreatment temperature and time), IL acid/base ratio and water content.

## Lignin characterisation

### Lignin subunit composition

**Fig. 7:**
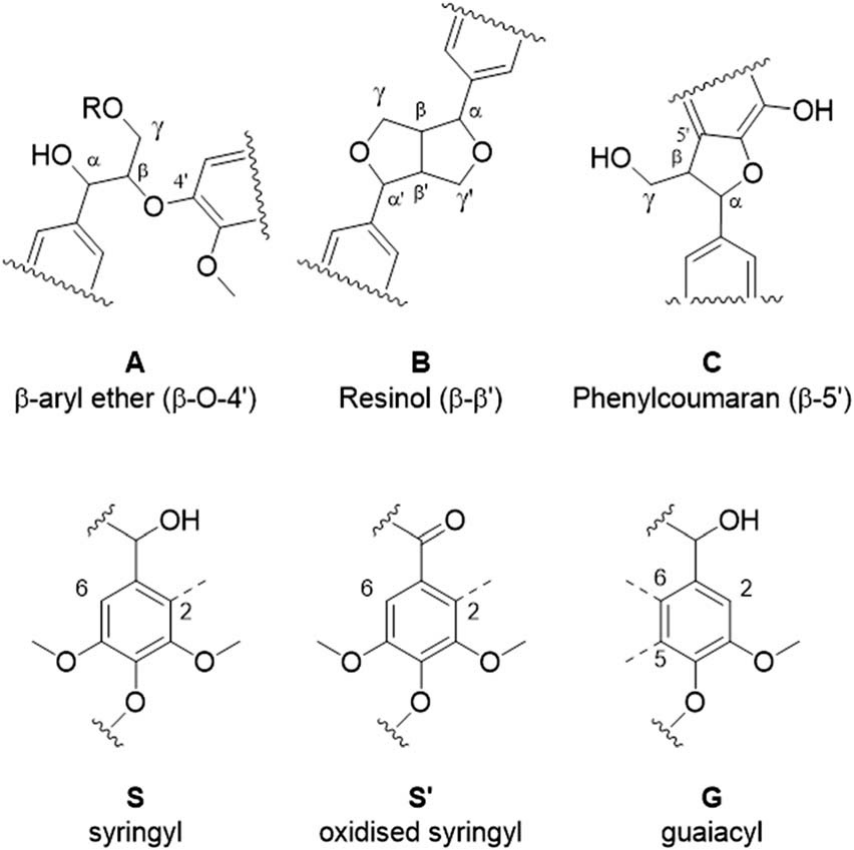
The most prominent ether bonds and lignin subunit in eucalyptus lignin.

Eucalyptus lignin from the time-course experiments at 120 and 150 °C was analysed using 2D HSQC NMR. Fig. 7 shows the chemical structures of the most important ether bonds and subunits found in eucalyptus lignin. Eucalyptus is a hardwood and as such its lignin contains both *S* and *G* units. *S*/*G* ratios of 3.12 to 3.67 were found for swollen residue enzyme lignin in a study looking at 1–4 year old trees while the most abundant ether linkages are the β-*O*-4′ and the β–β′ bonds, with *ca.* 60 and 10 linkages per 100 aromatic units, respectively.^61^ A different source reports large differences in *S*/*G* ratios of eucalyptus of different origins, the same origin, and even within the same tree, with values of the *S*/*G* ratio ranging from 3.5 to 6.5.^62^ Furthermore often different methods of analysis yield considerably different results,^63^ so caution is warranted and, especially using the semi-quantitative method of HSQC NMR, the numbers should not be taken at face value and instead the trends over a time course evaluated.

**Fig. 8:**
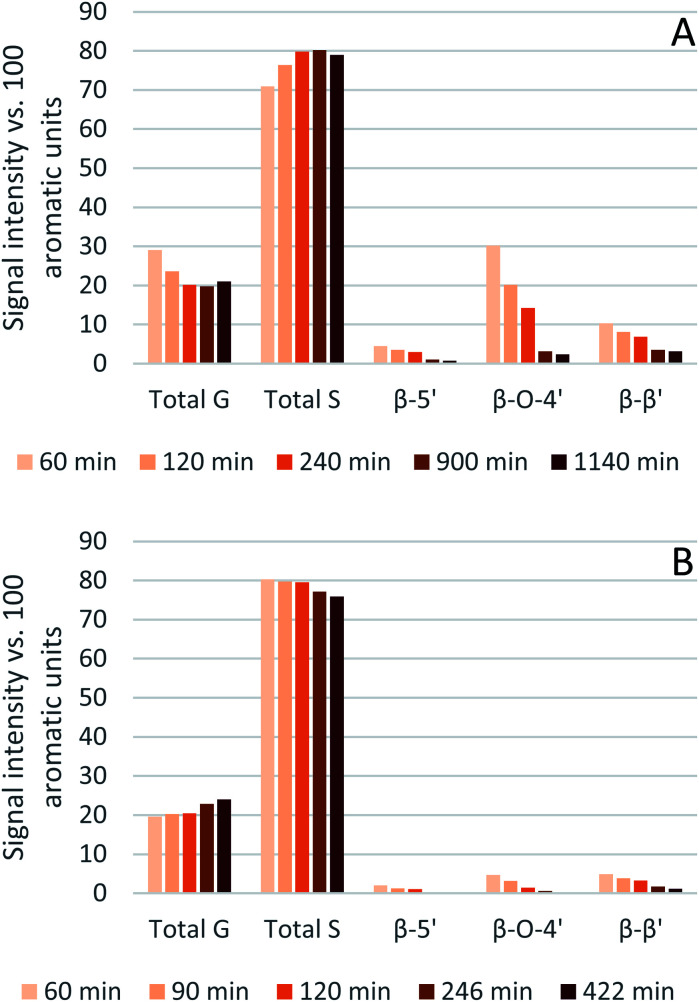
Abundance of S and G units and ether linkages of isolated eucalyptus lignin, obtained after pretreatment with [N_2220_][HSO_4_] with 20 wt% water at 120 °C (A) and 150 °C (B).

The results from the semi-quantitative HSQC analysis are shown in Fig. 8 and numerical results are given in Tables S7 and S8 (ESI†). The spectra (see ESI† for spectra) confirm that the β-*O*-4′ and the β–β′ bonds are the most abundant and, as seen in previous studies using triethylammonium hydrogen sulfate, the signals for the ether bonds disappear over time, indicating their cleavage or chemical modification.^33,64^ At 120 °C, the majority of the ether bonds appears to be broken or modified in the first 4 hours (240 min) of pretreatment, while at 150 °C, already after 60 min of pretreatment very few ether bond signals are detected. The amount of *S* compared to *G* in the precipitated lignin is seen to increase in the first 15 hours of pretreatment at 120 °C, with an *S*/*G* ratio of 2.4 after 1 h and 4.0 after 15 h (900 min), and to start decreasing thereafter. At 150 °C the amount of *S* relative to *G* decreases over the entire window of analysis from 4.1 after 1 hour (60 min) to 3.2 after 7 hours (422 min). With a reported *S*/*G* ratio for untreated eucalyptus lignins of 3 to over 6,^61,62^ the observed *S*/*G* ratio suggests that a relatively G-rich lignin is isolated initially, gradually becoming richer in *S* until *G* becomes enriched again. This trend of an initial increase in the *S*/*G* ratio followed by a levelling off or decrease has been seen in a previous study looking at willow, another hardwood, using the same ionic liquid.^28^ As a possible explanation for this observation it was proposed that S-rich lignin is more easily extracted out of the cell-wall as it is less cross-linked than G-rich lignin. Only harsher conditions (in this case longer pretreatment times) would lead to a lignin enriched in G. This however fails to explain why the *S*/*G* ratio initially is lower than what would be expected from the *S*/*G* ratio found for native lignin, and instead suggests that it is G-rich lignin that is extracted early on. A decrease over time in the *S*/*G* ratio in *Miscanthus* (grass) lignin has been reported using the protic ionic liquid 1-butylimidazolium hydrogen sulfate ([HC_4_im][HSO_4_]),^65^ however no explanation was proposed. A study from 1997 looking at S- and G-type model compounds under acidic cooking conditions found that ethers in the α-position of G-type units break faster than S-type linked ethers.^66^ The carbonium ions derived from the cleavage of these ethers undergo condensation with the electron-rich carbon on the aromatic units. The G-derived carbonium ions and the aromatic carbon on the S-type compounds showed the highest reactivity. Additionally, the diphenylmethane structures formed as a result of the condensation of the alpha carbonium ion with the aromatic carbon were more stable when formed with G aromatic carbons while the S derived diphenylmethane structures decomposed.^66^ These findings are reconciled with the observations from the present study: an initially low *S*/*G* ratio can be achieved as a result of the faster ether cleavage linked to G units, releasing the G unit more quickly into the ionic liquid. This is followed by an increase of the *S*/*G* ratio as most of the lignin is extracted, resulting in an observed *S*/*G* ratio which we can only assume to be reflective of the native lignin. At longer times, the S-derived condensation products start degrading, resulting in a reversal of the trend and a more G-rich lignin.

**Fig. 9:**
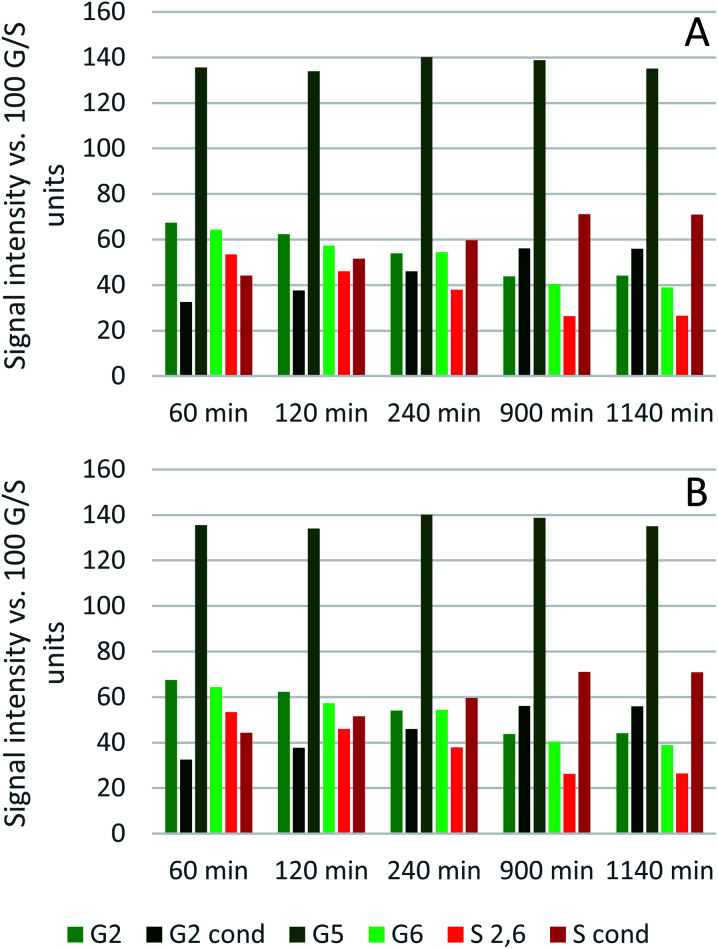
Abundance of C–H_*n*_ sites on the guaiacyl and syringyl rings of isolated eucalyptus lignin, obtained after pretreatment with [N_2220_][HSO_4_] with 20 wt% water at 120 °C (A) and 150 °C (B).

Comparing the S condensed with the total amount of *S* (as calculated by the sum of half the *S*_2,6_ and 
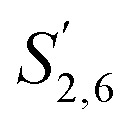
, plus *S*_cond_) and the various G signals with the total amount of *G* (as calculated by the sum of *G*_2_ and *G*_2,cond_), (Fig. 9) gives some insight on the progression of condensation reactions occurring on the two different lignin subunits. The G_2_ signal shifts to *G*_2,cond_ if other positions of the G unit are condensed. The positions that can undergo condensation are the 5 and 6 positions. Comparing their relative signal intensities over time shows that the G_5_ position is much less reactive than the G_6_ position, reflected in the decrease of G_6_ signal over the early periods of pretreatment. The G_5_ signal does not significantly change during the 19 h (1140 min) time course conducted at 120 °C and only starts decreasing after 2 hours at 150 °C. This is in line with what was previously observed for softwood lignin.^64^ The S signal shifts rapidly to *S*_cond_ within the first 15 h at 120 °C and the first 4 h (240 min) at 150 °C, confirming the observations from the model compound study that showed higher reactivity of the S aromatic carbons compared to the G aromatic carbons.^66^

### Molecular weight

Lignin molecular weight was analysed using Gel Permeation Chromatography (GPC). The results can be found in Table S9 (ESI†). IonoSolv lignin has in the past been proposed to go through four stages during the pretreatment:^64^ stage I where the lignin extraction has just started, resulting in high molecular weights (high *M*_w_ and *M*_n_, consequently low polydispersity index (PDI)) as the lignin has not been given enough time to depolymerise, followed by stage II where lignin extraction continues but some lignin has had time to depolymerise, resulting in lignin with still high *M*_w_ but a much decreased *M*_n_, reflected in a high PDI. Stage III is reached when lignin extraction has stopped, resulting in mostly depolymerised lignin with low *M*_w_ and low *M*_n_, and as a result again a low PDI. Stage IV is reached after prolonged treatment where condensation reactions lead to the formation of some high *M*_w_ fragments, resulting in an increased *M*_w_ while the *M*_n_ remains low, resulting in a higher PDI again.

The observations made here are roughly in line with this proposed model. At 120 °C, the *M*_w_ undergoes a sharp decline between 1 and 2 hours of pretreatment (stages I and II) and remains between 4.4 and 3.9 kDa for the next 13 h (780 min) (stage III), before increasing rapidly to over 6.3 kDa after 19 h (1140 min) (stage IV). At 150 °C already after 1 h the *M*_w_ is at its lowest at just over 3 kDa and rises from there to around 4200 after 4 h. No significant change is seen between 4 and 7 h (240 to 422 min). What is striking compared to *Miscanthus*^15,33^ and pine lignin^64^ analysed in the past, is the length of time the *M*_w_ of the eucalyptus lignin remains relatively low. The instability of the S-derived condensation product^66^ may contribute to this apparent stability of molecular weight if condensation and decomposition of the condensation product are in equilibrium. What causes the *M*_w_ to rise between 15 and 19 h (900 to 1140 min) of pretreatment at 120 °C is however unclear.

### Effect of CO_2_ pressure on lignin characteristics

Characterisation was carried out on the lignins obtained with different CO_2_ pressures. The HSQC NMR does not show significant changes between samples treated with different CO_2_ pressures (results in the ESI†). After 4 h (240 min) at 120 °C, the GPC results do not reveal any difference between the various samples. After 30 min at 120 °C the *M*_w_ found for the case without CO_2_ (L0) and 2.1 MPa (20 barg CO_2_) (LL) were both higher than seen for any of the other pretreatments at over 16 and 12 kDa, respectively. The 8.3 MPa (82 barg) 30 min (LH) sample could not be run on the GPC as it showed very poor solubility in the solvent, indicating the molecular weight was even higher than for the other two 30 min samples (L0 and LL). Whether there is a real difference between the different 30 min series (L0, LL and LH) or whether the lignin extracted at such short pretreatment time is of such high molecular weight that part of it becomes insoluble at some point during the GPC sample preparation, resulting in somewhat different results, is currently unclear.

## Experimental

### Materials

#### Synthesis of triethylammonium hydrogen sulfate [N_2220_][HSO_4_]

Triethylammonium hydrogen sulfate [N_2220_][HSO_4_] was synthesized using a PTFE lined batch reactor operated in a similar way as described by A. Cariglia.^67^ Briefly, triethylamine was added into the reactor simultaneously with H_2_SO_4_ (66.3 wt%), with dedicated peristaltic pump per chemical. The reactor was under stirring and cooled with a cooling fluid recirculated through an external jacket. The water content of 20 wt% was confirmed by Karl–Fischer titration (Mettler Toledo V20) in triplicate. For consistency, this same batch of IL solution was used for all pretreatment experiments. The acid : base ratio was not controlled.

#### CO_2_ experiments

**Fig. 10:**
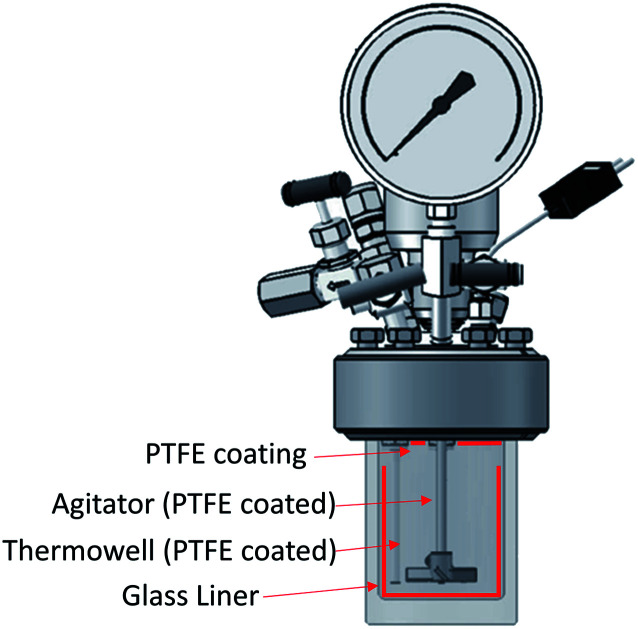
Schematic representation of the reactor used for the CO_2_ atmosphere experiments.

The pretreatments under CO_2_ atmospheres were conducted in a Parr reactor (Series Compact Reactor – PARR 5500 and a reactor controller Parr 4848). It has been noticed that the alkylammonium hydrogen sulfate ionic liquids can interact with stainless steels.^67^ To avoid any interference of the presence or metal in the pretreatment or reactor corrosion problems, a glass liner was used to hold the ionic liquid and biomass and all the wetted parts were protected with PTFE coatings and liners (Fig. 10). CO_2_ from a 5 kg CP grade CO_2_ DIP Tube cylinder from BOC was used (purity 99.995% pressure 5 MPa (50 bar)).

#### Feedstock

A plank of *Eucalyptus red grandis* was obtained from a local lumber (origin and age of the specimen unknown). It was air-dried, chopped and sieved (180–850 μm, 20 + 80 US mesh scale) prior to use and stored in plastic bags at room temperature in the dark.

#### Biomass fractionation and characterisation

The biomass fraction, moisture content, compositional analysis, delignification and hemicellulose removal, saccharification assay and biomass solubilisation are calculated using the formulas and experimental procedures described in our previous work.^15^ A detailed description of all procedures can be found in the ESI.†

#### Lignin characterisation

20 mg of isolated lignin was dissolved in 0.25 mL of DMSO-d^6^ and the solution transferred to a Shigemi tube. HSQC NMR spectra were recorded on a Bruker 600 MHz spectrometer (pulse sequence hsqcetgpsi,^2^ spectral width of 10 ppm in F2 (^1^H) with 2048 data points and 160 ppm in F1 (^13^C) with 256 data points, 16 scans and 1 s interscan delay). Spectra were analysed using MestReNova (Version 8.0.0, Mestrelab Research 2012). All spectra were referenced to the DMSO peak at 2.500 ppm (^1^H) and 39.520 ppm (^13^C). Integrals were obtained for spectra of the same series of experiments simultaneously to ensure that the same areas were integrated. All relevant spectra were copied into one file and selected them while integrating the relevant area in one spectrum. Integration areas were selected visually according to peak assignments found in literature.^65^ For ether linkages, the C–H_α_-signals were integrated. Integral sizes of 
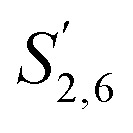
 and *S*_2,6_ were divided by two to account for the twice as large signal due to the symmetry in the S unit. Signals were reported with respect to 100 aromatic units calculated as:4
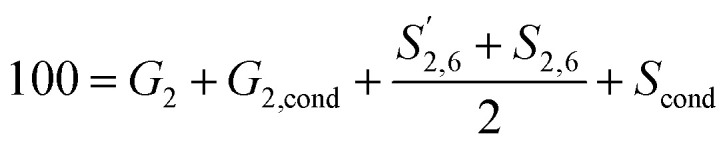


Additionally, different positions of the G unit were reported with respect to 100 G units calculated as:

5 100 = *G*_2_ + *G*_2,cond_

Condensation in *S* was described by reporting the different S signals with respect to 100 S units calculated as:6
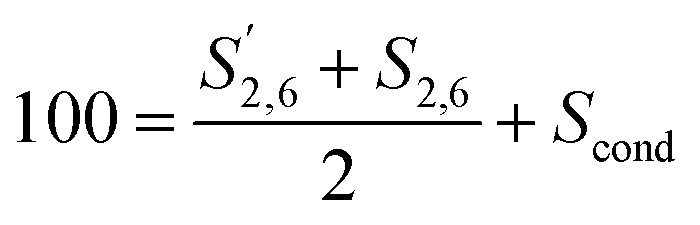


All spectra can be found in the ESI.†

## Conclusions

It has been demonstrated that the ionic liquid [N_2220_][HSO_4_] can be successfully applied for the pretreatment of *Eucalyptus red grandis*, to obtain cellulose-rich pulps with no hemicelluloses and low lignin contents. Additionally, these pretreated pulps have excellent enzymatic digestibility. Eucalyptus can be used as feedstock for cellulose-based materials, as well as biochemicals and biofuel production, after ionoSolv pretreatment.

Severities and H-factors seem promising tools for predicting process performance. However, generalised severity factors and detailed kinetic models, taking into account water content and acid : base ratio, need to be developed in order to perform process optimisations.

Changes in lignin structure were analysed. Eucalyptus lignin appears to undergo similar structural changes as other lignins during ionoSolv treatment, however with a lower degree of condensation occurring even after high severity treatments. This is potentially a benefit for further downstream processing as it will allow for a more consistent lignin output.

CO_2_-enriched atmospheres did not have a significant effect on pretreatment performance, nor in the properties of the pulp and lignin. This shows that the ionoSolv process is pressure insensitive in inert gases and CO_2_ up to 8.3 MPa (82 barg).

## Conflicts of interest

There are no conflicts to declare.

## Supplementary Material

RA-010-D0RA02040K-s001
