# Supplementary material for: Eucalyptus red grandis pretreatment with protic ionic liquids: effect of severity and influence of sub/super-critical CO2 atmosphere on pretreatment performance
Source: RSC Adv. 2020 Apr 22;10(27):16050–60. doi: 10.1039/d0ra02040k (PMC9052920; doi:10.1039/d0ra02040k)
Supplement: RA-010-D0RA02040K-s001 [file RA-010-D0RA02040K-s001.pdf]

Electronic Supplementary Material (ESI) for RSC Advances.
This journal is © The Royal Society of Chemistry 2020

Electronic Supplementary Information

Eucalyptus Red Grandis pretreatment with protic ionic liquid: Effect of severity and influence of sub/super-critical CO_2 atmosphere on pretreatment performance

Francisco Malaret,[a] Florence J. V. Gschwend,[a] Joana M. Lopes,[b] Wei-Chien Tu,[a] and Jason P. Hallett[a,*]

a. Department of Chemical Engineering, Imperial College London, London, SW7 2AZ, UK. E-mail: j.hallett@imperial.ac.uk; http://www.imperial.ac.uk/people/j.hallett; Tel: +44 (0) 2075945388

b. High Pressure Process Group, Department of Chemical Engineering and Environmental Technology, University of Valladolid, Spain.

1 Biomass fractionation and characterisation

The experimental procedures and formulas to calculate the moisture content, biomass fraction, compositional analysis, delignification and hemicellulose removal, saccharification assay and biomass solubilisation are described in this section.

1.1 Moisture content

For both raw and pretreated biomass the moisture content was determined according to the protocol by the National Renewable Energy Laboratory (NREL) "Determination of Total Solids in Biomass and Total Dissolved Solids in Liquid Process Samples"[1] by weighing out approximately 100 mg of biomass/pulp onto a preweighed piece of aluminium foil and recording the weight using the analytical balance. The foil with the biomass/pulp was folded and oven dried (T = 105 °C) overnight. The hot packets were placed in a desiccator to allow cooling to room temperature. The weight was recorded immediately, and the moisture content calculated.

$$\%Total\ Solids = \left(\frac{Weight_{Foil+oven\ dryed\ biomass} - Weight_{foil}}{Weight_{air-dryed\ biomass}}\right) \cdot 100\% \quad (1)$$

$$\%Moisture = 100 - \left(\frac{Weight_{Foil+oven\ dryed\ biomass} - Weight_{foil}}{Weight_{air-dryed\ biomass}}\right) \cdot 100\% \quad (2)$$

The moisture content of all Eucalyptus *Red Grandis* samples (raw and pretreated) ranged from 7% - 9%.

1.2 Fractionation of Biomass

The ionic liquid/water mixture was prepared by adding 20 wt% of water to the hydrogen sulfate ionic liquid triethylammonium hydrogen sulfate $[N_{2220}][HSO_4]$ and mixing until a homogenous solution was obtained. The water content was confirmed by Karl-Fischer titration in triplicate. The required amount of ionic liquid/water mixture was weighed into a 15 ml glass pressure tube with silicone front seal (Ace Glass) and the exact weight recorded. The air-dried biomass (Eucalyptus *Red Grandis*) was added to obtain a solvent ratio of 1:10 g/g. The containers were capped, and the content mixed with a vortex shaker. The samples were then placed into a preheated convection oven (OMH60 Heratherm Advanced Protocol Oven). After the pretreatment period, they were taken out and left to cool at room temperature. After the

pretreatment, 40 mL of ethanol was added to the pretreatment mixture and the suspension transferred into a 50 mL centrifuge tube. The tube was shaken for one minute and the mixture then left at room temperature for at least 1 hour. The tube was mixed again for 30 seconds and then centrifuged at 3500 rpm for 1 hour. The supernatant was decanted carefully into a round bottom flask. The washing step was repeated three more times. The remaining pulp was then transferred into a cellulose thimble and further washed by Soxhlet extraction with refluxing ethanol (150 mL) for 24 hours. The thimbles were then left on the bench overnight to dry. The ethanol used for the Soxhlet extraction was combined with the previous washes and evaporated under reduced pressure at 40 °C, leaving the dried ionic liquid/lignin mixture. To the dried ionic liquid/lignin mixture, 30 mL of water was added to precipitate the lignin. The suspension was then transferred into a 50 mL falcon tube, shaken for one minute and then left at room temperature for at least 1 hour. The tube was centrifuged and the supernatant decanted and collected in a round bottom flask. This washing step was repeated twice more. The air-dried pulp yield was determined by weighing the recovered biomass from the cellulose thimbles. The oven-dried yield was determined as described in section 1.1.

$$Yield_{pulp} = \left(\frac{Weight_{air-dryed\ biomass\ after\ pretreatment}}{Weight_{air-dryed\ biomass\ before\ pretreatment}}\right) \cdot \left(\frac{\%Moisture_{air-dryed\ biomass\ after\ pretreatment}}{\%Moisture_{air-dryed\ biomass\ before\ pretreatment}}\right) \cdot 100\% \tag{3}$$

The lid of the Falcon tube containing the lignin was removed. The tube was covered with a cellulose-paper, to avoid lignin contamination or losses and put into a vacuum oven overnight to dry at 40 °C under vacuum. The dried lignin was weighed the next day to calculate the lignin yields and delignification.

$$Lignin_{untreated} = Weight_{air-dryed\ untreted\ biomass} \cdot \left(\frac{100 - \%Moisture}{100}\right) \cdot \%Lignin_{untreated\ dry\ basis} \tag{4}$$

$$Yield_{lignin} = \frac{Weight_{dryed\ lignin\ after\ pretreatment}}{Weight_{air-dryed\ untreted\ biomass} \cdot \left(\frac{100 - \%Moisture}{100}\right)} \cdot 100\% \tag{5}$$

$$Recovery_{lignin} = \left(\frac{Weight_{dryed\ lignin\ after\ pretreatment}}{Lignin_{untreated}}\right) \cdot 100\% \tag{6}$$

$$Delignification = \left(\frac{Lignin_{untreated} - \left(Lignin_{pulp} \cdot Yield_{pulp}\right)}{Lignin_{untreated}}\right) \cdot 100\% \tag{7}$$

Where, $Lignin_{untreated}$ is the lignin content in untreated biomass (Eucalyptus *Red Grandis*), $Lignin_{pulp}$ is the lignin content in the pulp and $Yield_{pulp}$ is the oven-dried yield of pulp.

The experiments under CO_2 atmosphere were carried in a similar way. The biomass and ionic liquid were weighted in a glass liner, which was placed into the Parr reactor described in the experimental section of the main manuscript. The reactor was sealed according to the manufacturer instructions. The agitator of the reactor was used to mix the biomass and the IL, then it was switched off. The reactor was then pressurized with CO_2 to the required pressure. After the experiments, the reactor was let to cooled down to 50 °C and depressurised slowly by opening the depressuring valve. Once the reactor reached ambient temperature and

pressure, it was opened, the glass liner removed and the pretreated biomass washed as described previously.

A video describing the experimental protocol is available for consultation.[2]

1.3 Extractive determination

Ethanol extractives determination was carried out according to a published procedure by NREL "Determination of Extractives in Biomass" [3] in triplicates. Around 2.5 g were weighted and transferred into a cellulose thimble, which was placed into a Soxhlet extraction apparatus with refluxing ethanol (190 + 5 mL) for 24 hours. After this period, the system was allowed to cool down and the thimbles were washed with fresh ethanol and then left overnight to dry at room temperature. The solid were dried and the extractive content calculated:

$$\%Extractives = 100 - \left(\frac{Weight_{air-dryed\ extracted\ biomass}}{Weight_{air-dryed\ untreated\ biomass}}\right) \cdot 100\% \quad (8)$$

1.4 Compositional Analysis

Compositional analysis was carried out according to a published procedure by NREL "Determination of Structural Carbohydrates and Lignin in Biomass"[4]. 300 mg (on oven dry basis) of air-dry biomass after pretreatment and raw biomass without the extractive was weighed into a 100 mL pressure tube and the weight recorded. 3 mL of 72% sulfuric acid was added, the samples stirred with a Teflon stir rod and the pressure tubes placed into a preheated water bath at 30 °C. The samples were stirred again every 15 min for one hour, they were then diluted with 84 mL distilled water and sealed. The samples were autoclaved (Sanyo Labo Autoclave ML5 3020 U) for one hour at 121 °C and left to cool. The samples were then filtered through filtering ceramic crucibles of a known weight. The filtrate was stored in two plastic tubes and the remaining residue washed with distilled water. The crucibles were placed into a convection oven (VWR Venti-Line 115) at 105 °C for 24±2 hours. They were placed in a desiccator for 15 min and the weight recorded. The crucibles were then placed into a muffle oven (Nabertherm + controller P 330) and ashed to constant weight at 575 °C. The crucible weight after ashing was recorded. The content of acid insoluble lignin (AIL) was determined according to Eq. 9.

$$\%\mathrm{AIL} = \frac{\mathrm{Weight}_{\mathrm{crucible+\ AIR}} - \mathrm{Weight}_{\mathrm{crucible+\ ash}}}{\mathrm{Weight}_{\mathrm{oven-dry\ sample}}} \cdot 100 \quad (9)$$

where $\mathrm{Weight}_{\mathrm{crucibles+AIR}}$ is the weight of the oven-dried crucibles plus the acid insoluble residue, $\mathrm{Weight}_{\mathrm{crucibles+ash}}$ is the weight of the crucibles after ashing to constant temperature at 575 °C. The supernatant was used for the determination of acid soluble lignin content (ASL) by UV analysis at 240 nm (Eq. 10) using a Perkin Elmer Lambda 650 UV/Vis spectrometer.

$$\%\mathrm{ASL} = \frac{A}{l \cdot \varepsilon \cdot c} \cdot 100\% = \frac{A \cdot V_{\mathrm{filtrate}}}{l \cdot \varepsilon \cdot \mathrm{Wegiht}_{\mathrm{oven-dry\ sample}}} \cdot 100\% \quad (10)$$

Where A is the absorbance at 240 nm, l is the path length of the cuvette in cm (1 cm in this case), ε is the extinction coefficient (25 L/g cm), c is the concentration in mg/mL, the oven-dried weight of the sample in mg and $V_{filtrate}$ is the volume of the filtrate in mL and equal to 86.73 mL.

Calcium carbonate was added to the second liquid fraction until pH 5 was reached. The liquid was passed through a 0.2 µm PTFE syringe filter and subsequently submitted to HPLC analysis (Shimadzu, Aminex HPX-97P from Bio-Rad, 300 x 7.8 mm, purified water as mobile phase at

0.6 ml/min, column temperature 85 °C, de-ashing columns were used as pre-filters) for the determination of total sugar content. Calibration standards with concentrations of 0.1, 1, 2 and 4 mg/mL of glucose, xylose, mannose, arabinose and galactose were used. Sugar recovery standards were prepared as 10 mL aqueous solutions close to the expected sugar concentration of the samples and transferred to pressure tubes. 278 µL 72% sulfuric acid was added, the pressure tube closed and autoclaved and 3 the sugar content determined as described above. The sugar recovery coefficient (SRC) was determined according to Eq. 11 and the sugar content of the analysed sample using Eq. 12.

$$\mathrm{SRC} = \frac{C_{HPLC} \cdot V}{initial\ weight} \tag{11}$$

$$\%\mathrm{Sugar} = \frac{C_{HPLC} \cdot V \cdot corr_{anhydro}}{SRC \cdot \mathrm{Weight}_{\mathrm{oven-dry\ sample}}} \cdot 100\% \tag{12}$$

where c_{HPLC} is the sugar concentration detected by HPLC, V is the initial volume of the solution in mL (10.00 mL for the sugar recovery standards and 86.73 mL for the samples), initial weight is the mass of the sugars weighed in, $corr_{anhydro}$ is the correction for the mass increase during hydrolysis of polymeric sugars (0.90 for the C6 sugars glucose, galactose and mannose and 0.88 for the C5 sugars xylose and arabinose) and the oven-dried weight of the sample in mg.

1.5 Saccharification Assay

Saccharification assays were carried out according to a protocol published by the NREL "Low Solids Enzymatic Saccharification of Lignocellulosic Biomass"[5] in triplicate with blanks (also in triplicate). All reagents were purchased from Sigma Aldrich. The detailed procedure is as follows:

100±10 mg (ODW basis) of air-dried was placed into a Sterilin tube and the weight recorded. Three enzyme only blanks were run with 100 µL of purified water instead of biomass in order to correct for sugar residues present in the enzyme solutions. 9.9 mL solution made from 5 mL 100mM sodium citrate buffer at pH 4.8, 40 µL tetracycline solution (10 mg/mL in 70% ethanol), 30 µL cycloheximide solution (10 mg/mL in purified water), 4.71 mL purified water and 50 µL of Novozymes experimental enzyme mixture NS-22201 was added, the tubes closed and placed into an Stuart Orbital Incubator (S1500) at 50 °C and 250 rpm for 7 days at 50°C and 250 rpm. End point samples were obtained by filtering 1 mL of the saccharification mixture through a PTFE syringe filter. Samples were analysed on Shimadzu HPLC system with RI detector and an Aminex HPX87P column (BioRad, 300 x 7.8 mm) with purified water as mobile phase (0.6 mL/min). The column temperature was 85°C and acquisition time was 40 min. Calibration standards with concentrations of 0.1, 1, 2 and 4 mg/mL of glucose, xylose, mannose, arabinose and galactose and 8 mg/mL of glucose were used.

2 Additional Figures

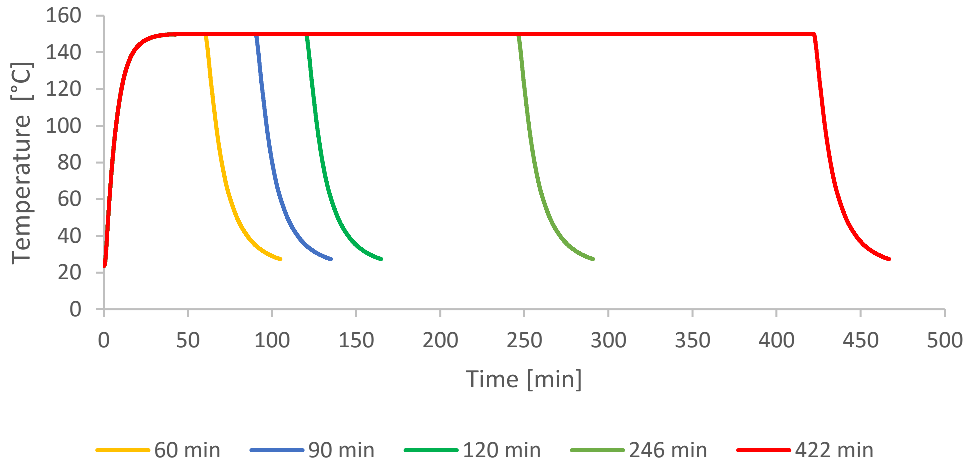

Figure S1. Typical reaction medium temperature inside an unstirred reaction tube (15 ml) at an oven temperature of 150°C for different pretreatments times.

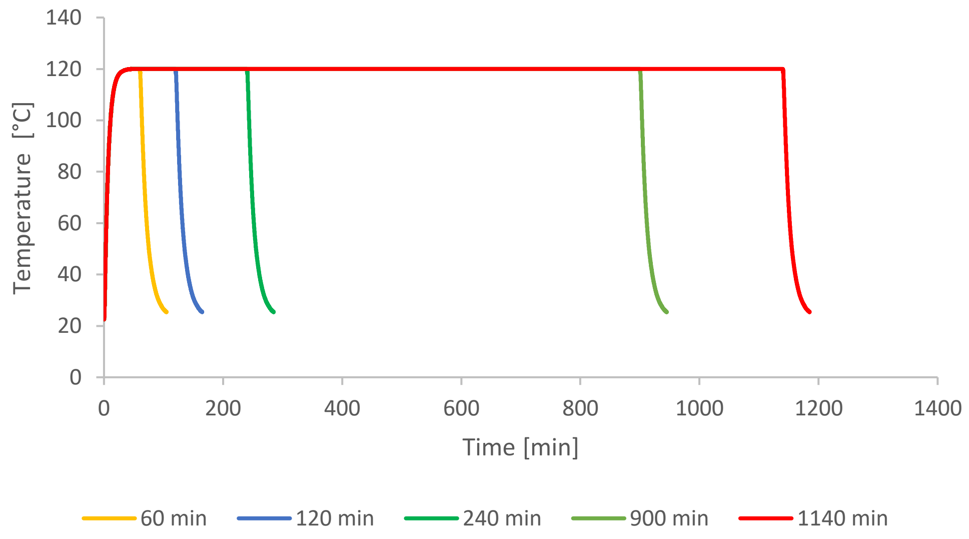

Figure S2 Typical reaction medium temperature inside an unstirred reaction tube (15 ml) at an oven temperature of 120°C for different pretreatments times.

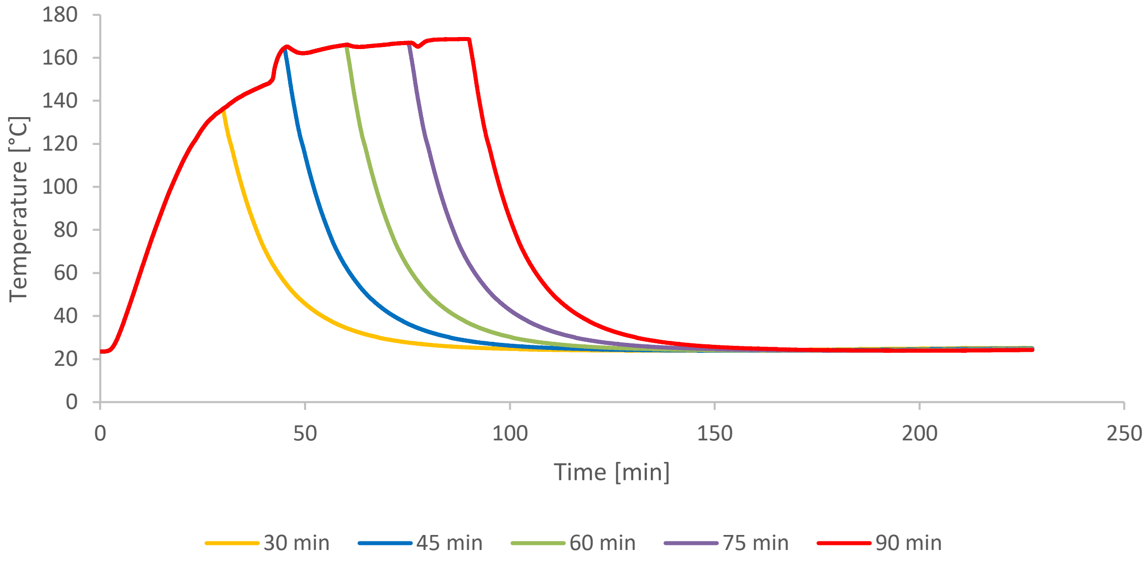

Figure S3 Typical reaction medium temperature inside an unstirred reaction tube (100 ml) at an oven temperature of 170°C for different pretreatments times.

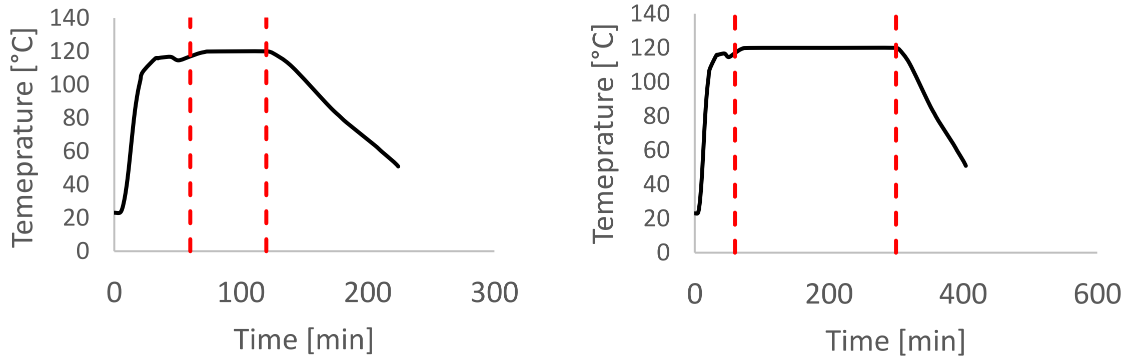

Figure S4. Temperature profile inside the high pressure reactor. Right: 30 min pre-treatment (low). Left: 4h pre-treatment (high)

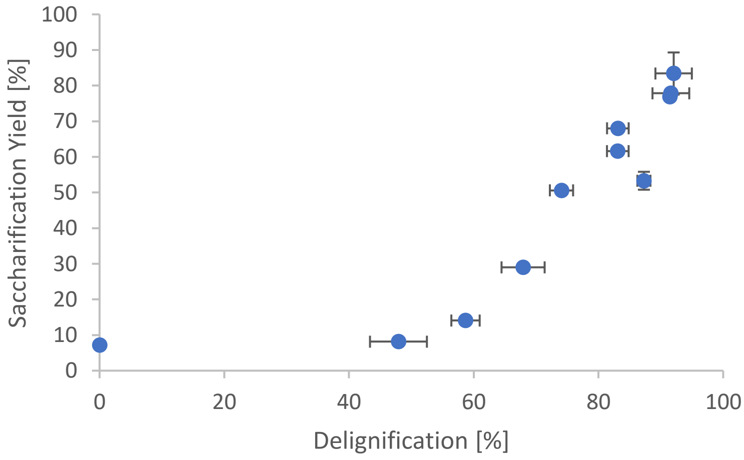

Figure S5. Correlation of enzymatic glucose release and delignification of Eucalyptus after pretreatment with [N_{2220}][HSO_4] with a water content of 20 wt% and a solid to solvent ratio of 1 : 10 g g^{-1} at different temperatures and times. Percentage of the theoretical maximum according to compositional analysis is shown.

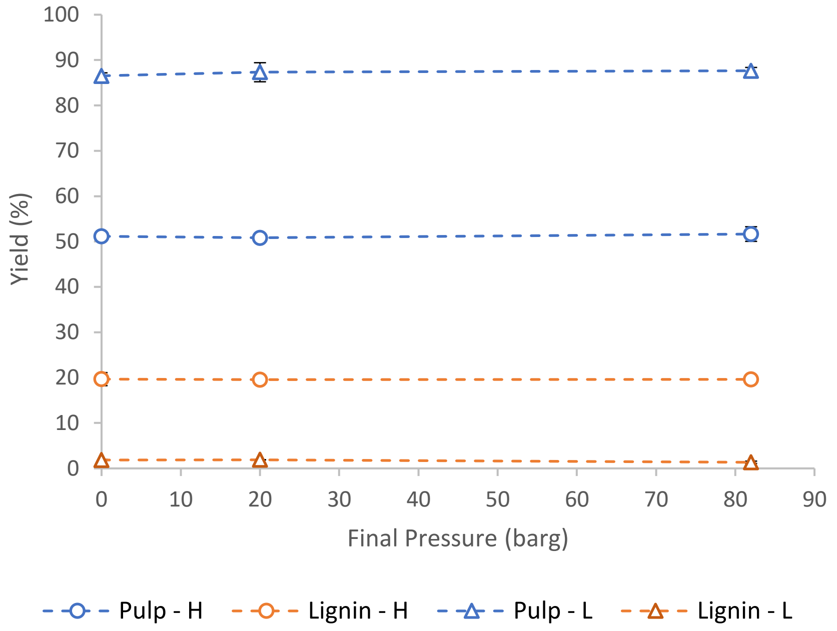

Figure S6. Pulp and lignin yields for the experiments carried out under CO2 atmospheres. H = 240 min pretreatment time and L = 30 min pretreatment times.

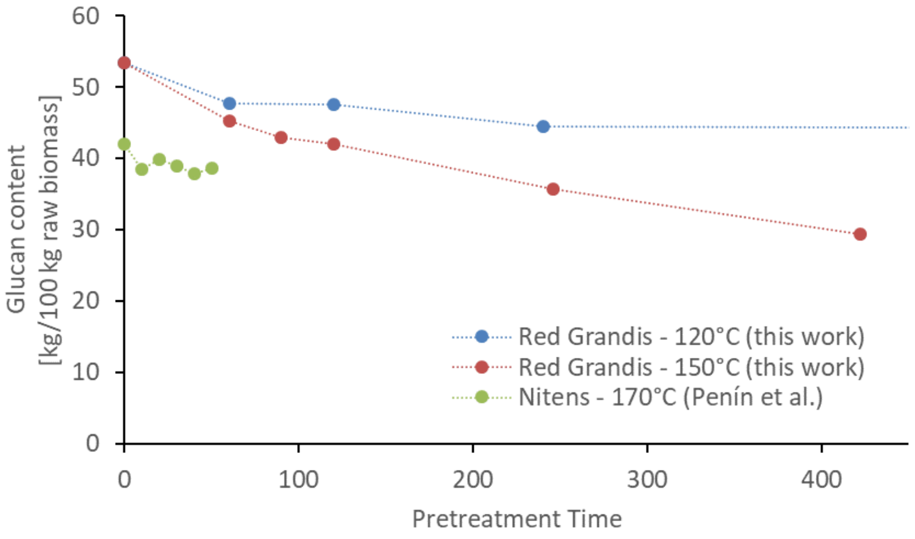

Figure S7. Comparison of glucan content of 2 eucalyptus species pretreated with $[N_{2220}][HSO_4]$.

3 Additional tables

Table S1. Calculated severity parameters and H-factors for the temperature profiles shown in Figures S1 and S2), assuming isothermal conditions and the respective error.

Pretreatment		R_0			H-Factors		
Time [min]	Temperature [°C]	Calculated	Isothermal Equivalent	Error (%)	Calculated	Isothermal Equivalent	Error (%)
60	120	181	233	22.2	391	547	28.6
120	120	414	466	11.1	938	1094	14.3
240	120	880	931	5.5	2032	2189	7.2
900	120	3441	3492	1.5	8052	8209	1.9
1140	120	4372	4424	1.2	10241	10398	1.5
60	150	1298	1780	27.1	6876	10006	31.3
90	150	2187	2669	18.1	11879	15009	20.9
120	150	3077	3559	13.5	16882	20012	15.6
246	150	6815	7297	6.6	37894	41024	7.6
422	150	12035	12517	3.9	67245	70374	4.4

Table S2. CO_2 experimental matrix.

		Pretreatment Time	
		Low [30 min]	High [240 min]
Pressure	Low [2.1±0.2 MPa (20±2 barg)]	LL	HL
	High [8.3±0.2 MPa (82±2 barg)]	LH	HH

Table S3. Pulp composition, as determined by compositional analysis. Average of replicates.

Pretreatment conditions	Composition wt%						
	%Glucan	%Xylan	%ASL	%AIL	%Lignin	%Ash	Extractive
Untreated	50.3	10.0	5.2	28.1	33.3	-	0.7
120°C - 60 min	74.9	10.6	4.0	24.8	28.8	-	-
120°C - 120 min	79.3	3.8	3.4	20.9	24.3	-	-
120°C - 240 min	89.9	2.2	2.3	16.2	18.5	-	-
120°C - 900 min	98.3	-	1.5	11.8	13.3	-	-
120°C - 1140 min	112.2	-	1.1	14.2	6.5	-	-
150°C - 60 min	105.4	-	1.6	4.9	6.5	-	-
150°C - 90 min	91.9	-	1.2	5.1	6.3	-	-
150°C - 120 min	91.9	-	1.2	5.4	6.6	-	-
150°C - 246 min	99.4	-	1.3	11.2	12.5	-	-
150°C - 422 min	78.6	-	1.2	29.1	30.3	-	-
L0	56.9	8.1	4.5	25.0	29.5	-	-
LL	56.4	7.8	4.4	25.5	29.8	-	-
LH	54.2	8.6	4.5	25.2	29.7	-	-
H0	70.3	-	1.3	7.1	8.3	-	-
HL	88.8	-	1.0	8.0	9.0	-	-
HH	87.8	-	1.0	7.3	8.2	-	-

Table S4. Normalized average pulp composition.

Pretreatment conditions	Composition wt%						
	%Glucan	%Xylan	%ASL	%AIL	%Lignin	%Ash	Extractive
Untreated	53.4	10.6	5.5	29.8	35.3	-	0.7
120°C - 60 min	65.5	9.3	3.5	21.7	25.2	-	-
120°C - 120 min	73.9	3.5	3.1	19.5	22.6	-	-
120°C - 240 min	81.3	2.0	2.1	14.6	16.7	-	-
120°C - 900 min	88.1	-	1.3	10.6	11.9	-	-
120°C - 1140 min	88.0	-	0.9	11.2	5.1	-	-
150°C - 60 min	94.2	-	1.4	4.4	5.8	-	-
150°C - 90 min	93.6	-	1.2	5.2	6.4	-	-
150°C - 120 min	93.3	-	1.2	5.5	6.7	-	-
150°C - 246 min	88.8	-	1.1	10.0	11.2	-	-
150°C - 422 min	72.2	-	1.1	26.7	27.8	-	-
L0	60.2	8.6	4.8	26.4	31.2	-	-
LL	60.0	8.3	4.7	27.1	31.7	-	-
LH	58.6	9.3	4.8	27.3	32.1	-	-
H0	89.4	-	1.6	9.0	10.6	-	-
HL	90.8	-	1.0	8.1	9.2	-	-
HH	91.4	-	1.0	7.6	8.6	-	-

Table S5. Standard deviation in pulp composition determined by compositional analysis.

Pretreatment conditions	Composition errors (wt%)						
	%Glucan	%Xylan	%ASL	%AIL	%Lignin	%Ash	Extractive
Untreated	0.3	0.04	0.1	0.4	0.5	-	0.05
120°C - 60 min	1	0.06	0.1	6	6	-	-
120°C - 120 min	3	0.8	0.07	0.5	0.6	-	-
120°C - 240 min	3	0.8	0.1	0.5	0.5	-	-
120°C - 900 min	3	0.8	0.1	0.5	0.5	-	-
120°C - 1140 min	4	-	0.1	0.5	0.5	-	-
150°C - 60 min	4	-	0.1	1.0	1	-	-
150°C - 90 min	0.2	-	0.01	1	1	-	-
150°C - 120 min	4	-	0.1	0.1	0.1	-	-
150°C - 246 min	4	-	0.1	0.3	0.3	-	-
150°C - 422 min	4	-	0.1	5	5	-	-
L0	0.4	0.04	0.05	0.1	0.1	-	-
LL	2	0.6	0.06	0.7	0.8	-	-
LH	0.8	0.8	0.04	0.4	0.4	-	-
H0	2	0	0.4	1	2	-	-
HL	0.2	0	0.02	0.3	0.3	-	-
HH	0.6	0	0.03	0.3	0.3	-	-

Table S6. Pretreatment performance

Pretreatment conditions	Pulp Yield (%)		Delignification (%)		Glucan Recovery (%)		Hemicellulose Removal (%)		Lignin Recovery (%) (*)		Lignin Yield (%) (**)	
	Value	Error	Value	Error	Value	Error	Value	Error	Value	Error	Value	Error
120°C - 60 min	72.9	0.3	48	9	89	3	35.8	0.8	11.4	0.5	4.0	0.2
120°C - 120 min	64	2	59	2	89	6	79	7	24	1	8.3	0.4
120°C - 240 min	55	2	74	2	83	6	90	7	60	6	21	1
120°C - 900 min	49.9	0.7	83	2	82	6	100	-	85	3	30	1
120°C - 1140 min	49.6	0.7	83	2	82	7	100	-	92	2	32.5	0.7
150°C - 60 min	48.1	0.7	92	3	85	7	100	-	66	4	23	1
150°C - 90 min	45.9	0.4	92	3	80.5	0.8	100	-	77	3	27	1
150°C - 120 min	45.1	0.2	91.5	0.5	79	7	100	-	86	14	31	5
150°C - 246 min	40	2	87	1	67	7	100	-	73	3	26	1
150°C - 422 min	41	3	67.9	6.9	55	7	100	-	66	13	23	5
L0	86.5	0.6	8.3	0.1	97	1	29.7	0.3	5.2	0.4	1.9	0.1
LL	87	2	7.6	0.4	98	5	31	3	5.4	0.1	1.89	0.02
LH	87.6	0.7	7.2	0.2	96	2	23	2	3.8	0.8	1.35	0.26
H0	51.2	0.3	30	5	86	2	100	-	56	5	20	1
HL	50.8	0.4	31	1	86.4	0.9	100	-	55	2	19.6	0.6
HH	52	2	31	2	88	3	100	-	56	2	19.6	0.3

(*) Relative to the total lignin amount in untreated biomass.

(**) Relative to the total biomass (dry basis).

Table S7. HSQC Integrals normalised to 100 S+G units for the isolated Eucalyptus lignins.

	**120°C 60 min**	**120°C 120 min**	**120°C 240 min**	**120°C 900 min**	**120°C 1140 min**	**150°C 60 min**	**150°C 90 min**	**150°C 120 min**	**150°C 246 min**	**150°C 422 min**
**G_2**	19.6	14.7	10.9	8.7	9.3	9.5	9.1	9.1	9.8	10.3
**$G_{2,cond}$**	9.4	8.9	9.3	11.1	11.8	10.1	11.1	11.4	13.0	13.7
**G_5**	39.4	31.6	28.3	27.5	28.4	27.8	29.0	29.2	30.1	29.9
**6**	18.7	13.5	11.0	8.0	8.2	9.1	8.9	8.1	8.6	9.0
**$S'_{2,6}$**	1.7	1.9	2.0	2.0	2.1	2.3	2.3	2.4	2.0	1.8
**$S_{2,6}$**	37.9	35.2	30.2	21.1	20.9	25.2	22.9	22.1	19.1	17.7
**S_{cond}**	31.4	39.4	47.6	57.0	55.9	52.8	54.6	55.1	56.0	56.5
**Total G**	29.0	23.6	20.2	19.8	21.1	19.6	20.2	20.5	22.8	24.0
**Total S**	71.0	76.4	79.8	80.2	78.9	80.4	79.8	79.5	77.2	76.0
**β-5'**	4.5	3.6	3.0	1.0	0.8	2.0	1.3	1.0	0.3	0.1
**β-O-4'**	30.3	20.2	14.3	3.2	2.4	4.7	3.1	1.4	0.6	0.3
**β-β'**	10.3	8.2	6.9	3.5	3.2	4.9	3.9	3.2	1.8	1.2
**S/G ratio**	2.4	3.2	4.0	4.0	3.8	4.1	3.9	3.9	3.4	3.2

Table S8 Results of the HSQC analysis of Eucalyptus lignin isolated with under varying severities and CO_2 pressures. Numbers are reported relative to 100 G+S units.

	**L0**	**LL**	**LH**	**H0**	**HL**	**HH**
**G_2**	66.0	67.0	66.2	45.5	45.3	44.8
**$G_{2,COND}$**	34.0	33.0	33.8	54.5	54.7	55.2
**G_6**	64.0	61.9	61.9	45.6	45.6	45.9
**G_5**	106.1	104.2	105.4	108.1	105.2	109.7
**$S'_{2,6}$**	3.0	2.9	2.8	3.1	3.1	3.3
**$S_{2,6}$**	67.7	67.6	68.7	36.3	36.2	35.2
**S_{COND}**	29.3	29.5	28.5	60.6	60.7	61.5

Table S9 Results from the gel permeation chromatography. *It was not possible to analyse the lignin of sample LH as the lignin did not sufficiently dissolve in the solvent.

**Pretreatment conditions**		**M_n [Da]**	**M_w [Da]**	**PDI**
120°C	60 min	1708	5259	3.1
120°C	120 min	1397	4344	3.1
120°C	240 min	1553	4181	2.7
120°C	900 min	1249	3978	3.2
120°C	1140 min	1296	6384	4.9
150°C	60 min	1129	3029	2.7
150°C	90 min	1137	3178	2.8
150°C	120 min	938	3362	3.6
150°C	246 min	1243	4168	3.4
150°C	422 min	1201	4234	3.5
L0		2554	16132	6.3
LL		2561	12130	4.7
LH*		-	-	-
H0		1284	4126	3.2
HL		1324	4272	3.2
HH		1325	4387	3.3

4 ANOVA Analysis (Saccharification yields of IL pretreatment under CO_2 atmospheres)

	Saccharification Yield (% of theoretical maximum)	
	Experiment 1	Experiment 2
H0	82.59	82.51
HL	84.78	85.46
HH	86.89	82.48
L0	7.56	7.28
LL	7.77	7.40
LH	6.93	7.12

ANOVA – High severity (pretreatment time 240 min)

Groups	*Count*	*Sum*	*Average*	*Variance*
H0	2	165.0966	82.54831	0.002841
HL	2	170.2453	85.12267	0.229667
HH	2	169.3778	84.68892	9.719482

Source of Variation	*SS*	*df*	*MS*	*F*	*P-value*	*F crit*
Between Groups	7.598446	2	3.799223	1.145265	0.427005	9.552094
Within Groups	9.951991	3	3.31733			
Total	17.55044	5				

ANOVA – High severity (pretreatment time 30 min)

Groups	*Count*	*Sum*	*Average*	*Variance*
L0	2	14.84474	7.42237	0.040372
LL	2	15.17159	7.585795	0.069103
LH	2	14.04833	7.024163	0.017522

Source of Variation	*SS*	*df*	*MS*	*F*	*P-value*	*F crit*
Between Groups	0.333804	2	0.166902	3.942683	0.144683	9.552094
Within Groups	0.126996	3	0.042332			
Total	0.4608	5				

5 Lignin HSQC Spectra

5.1 120°C, 60 min (1 h)

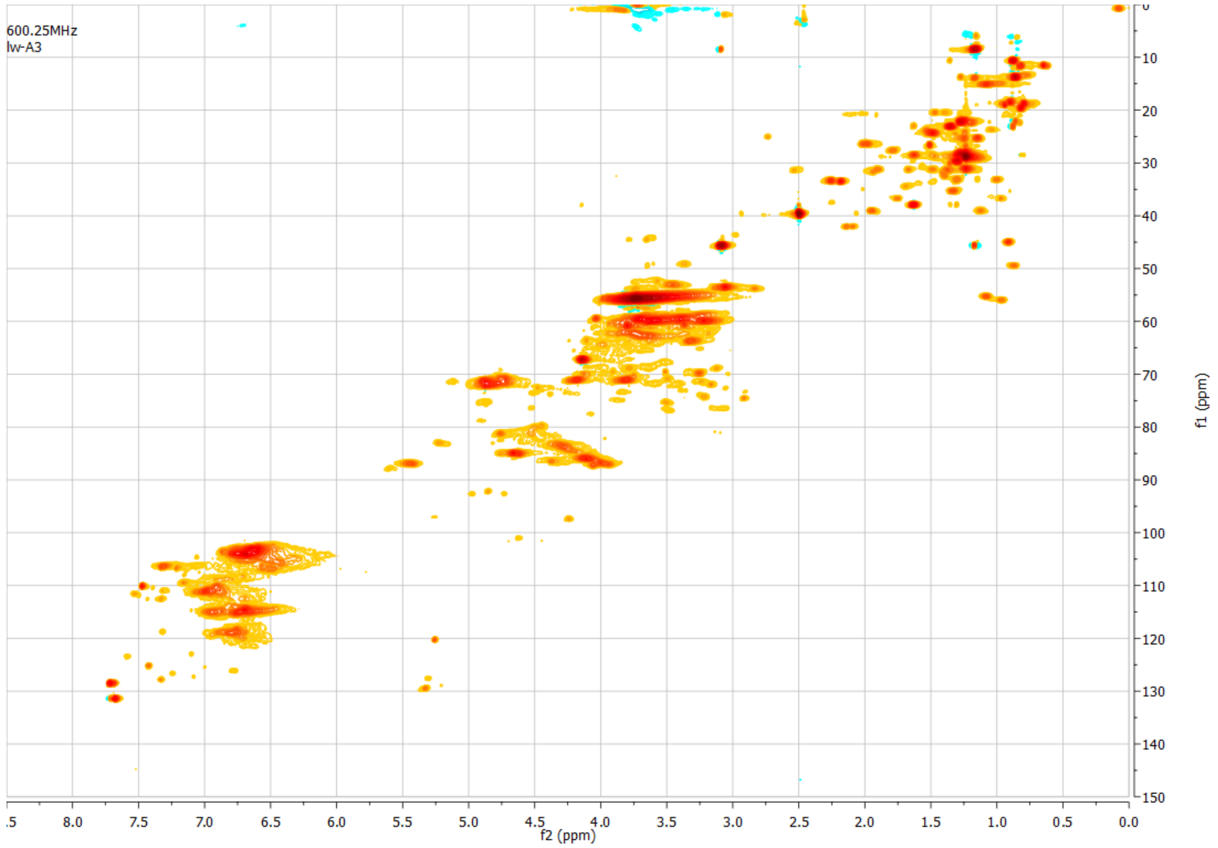

5.2 120°C, 240 min (2 h)

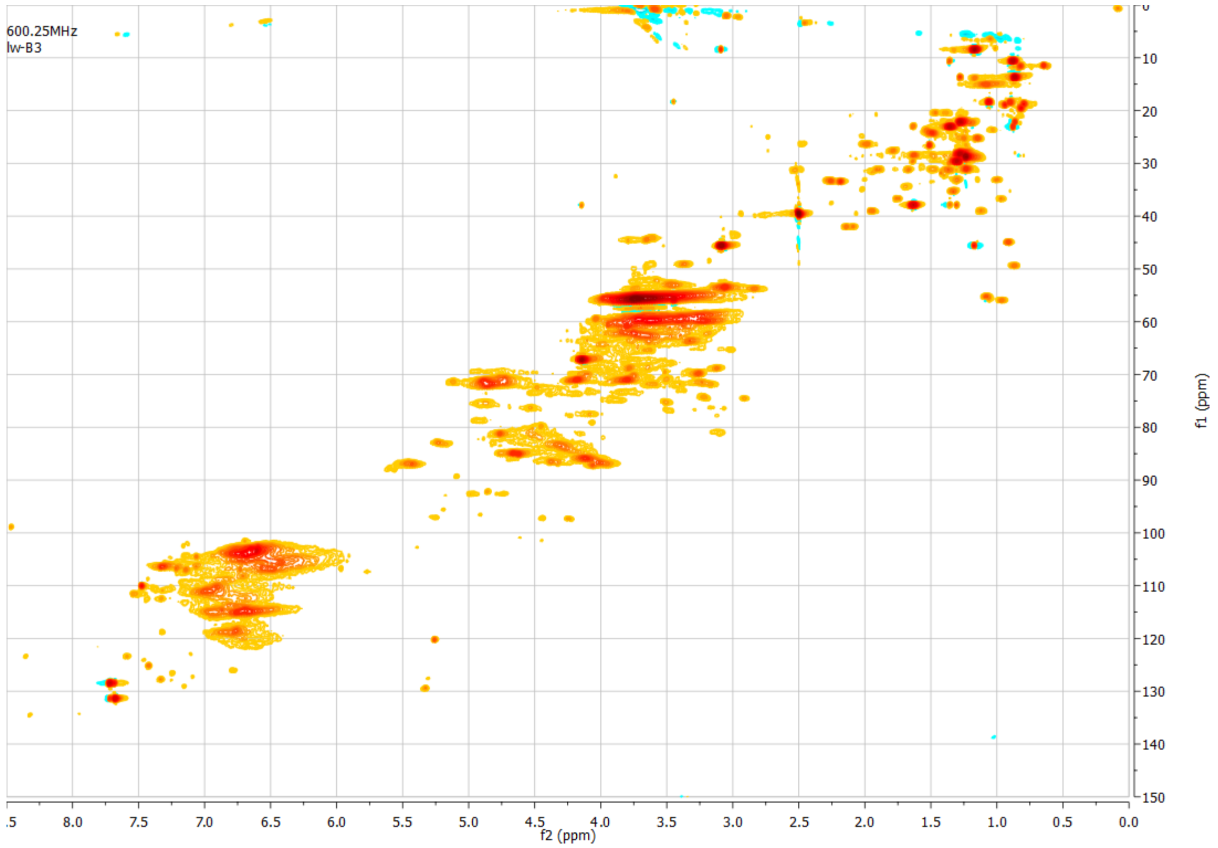

5.3 120°C, 240 min (4 h)

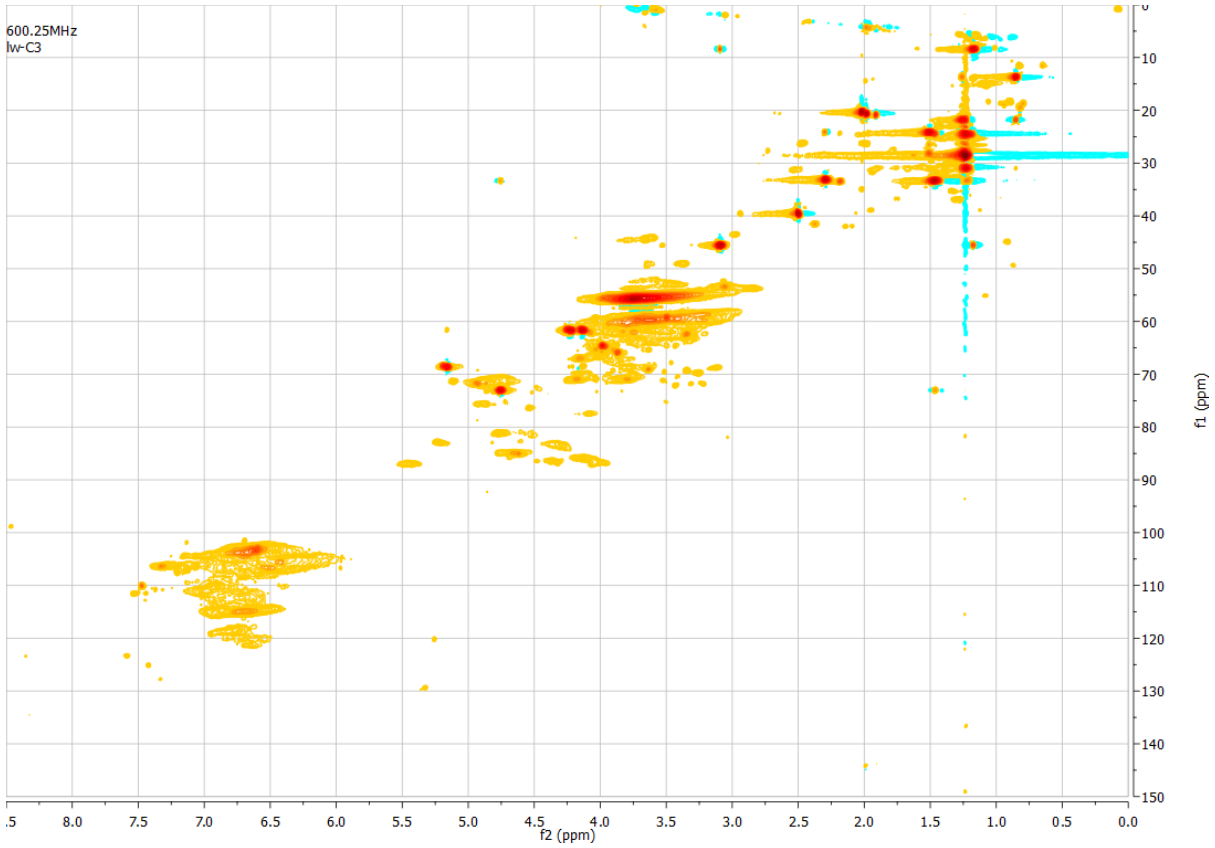

5.4 120°C, 900 min (15 h)

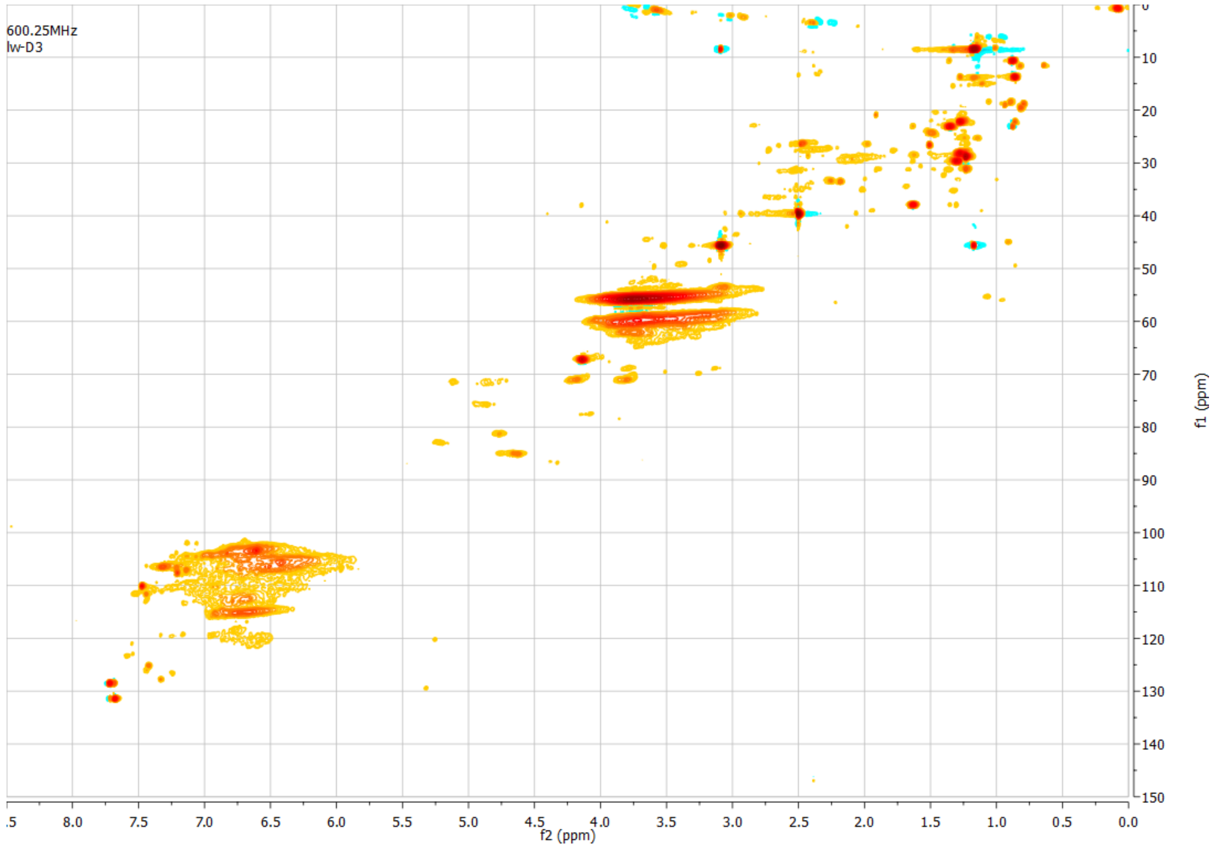

5.5 120°C, 1140 min (19 h)

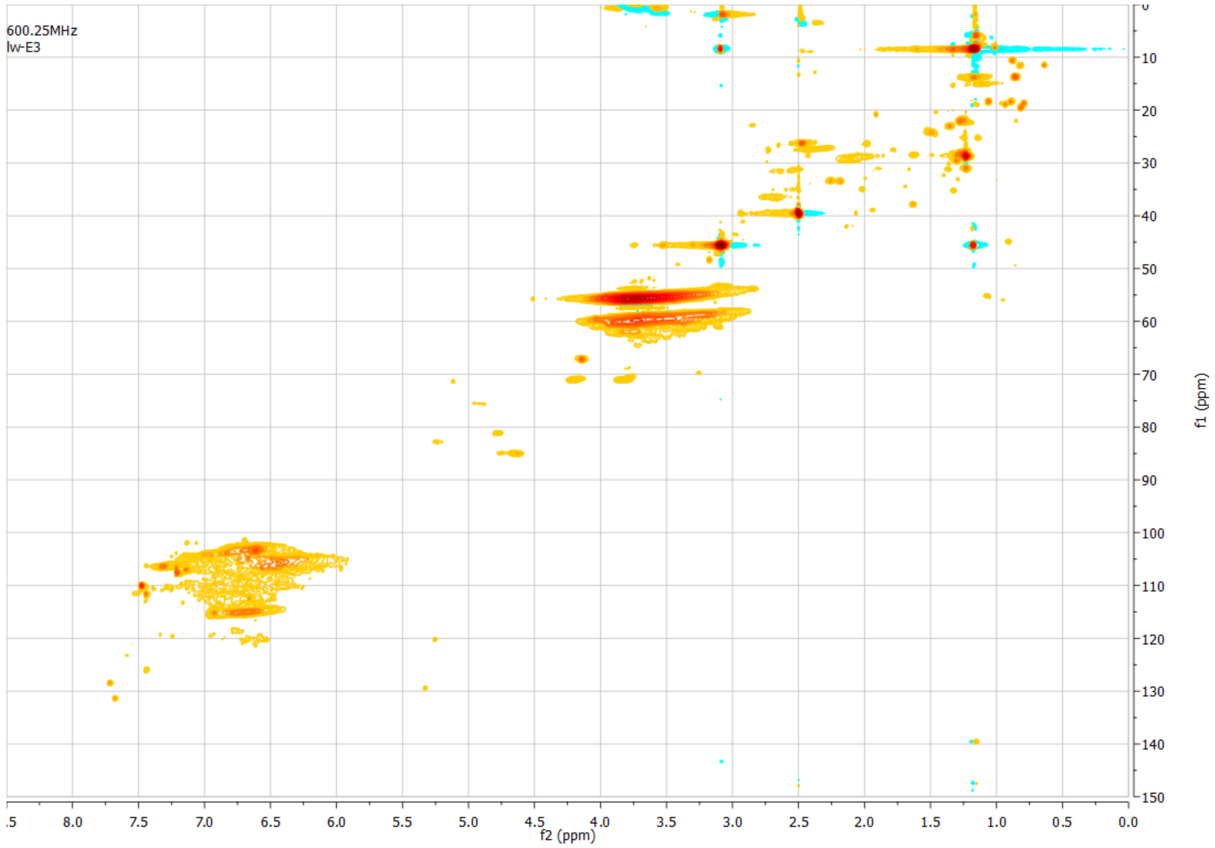

5.6 150°C, 60 min (1 h)

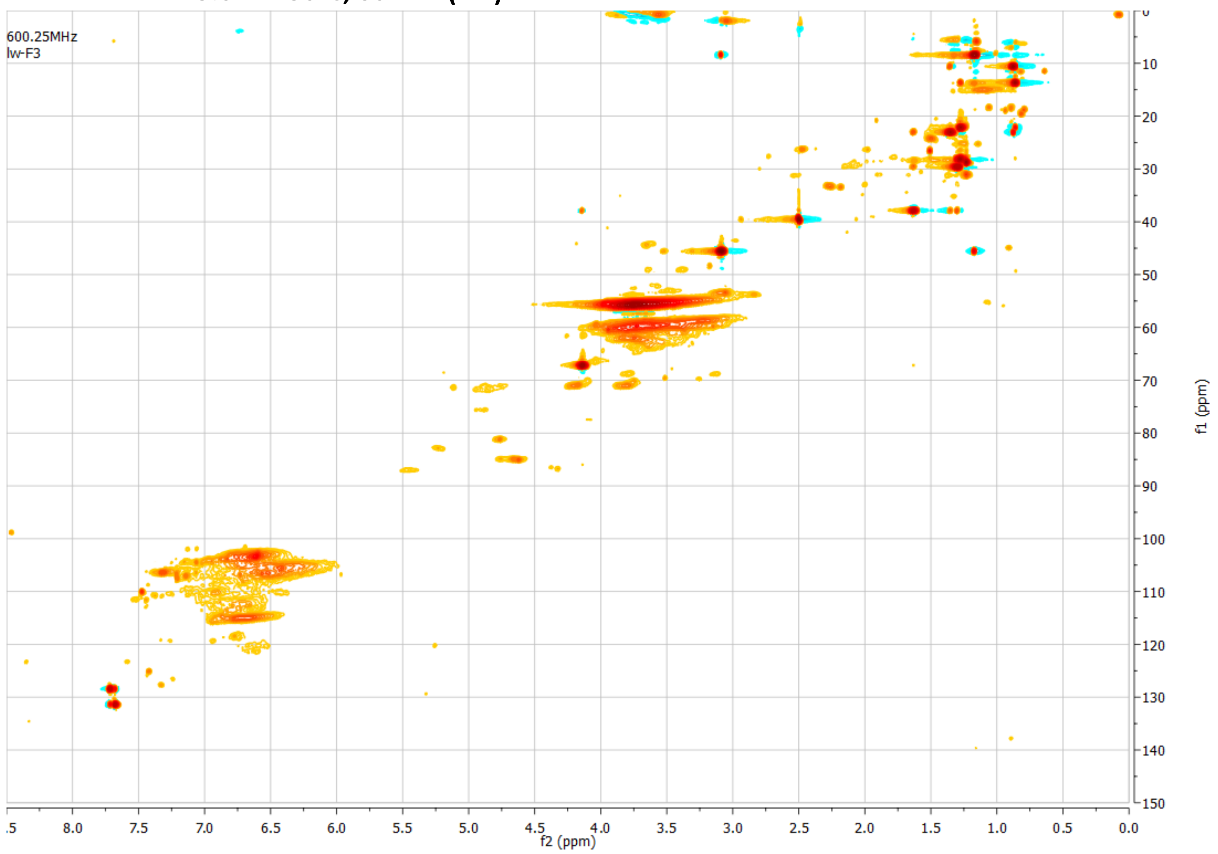

5.7 150°C, 90 min (1.5 h)

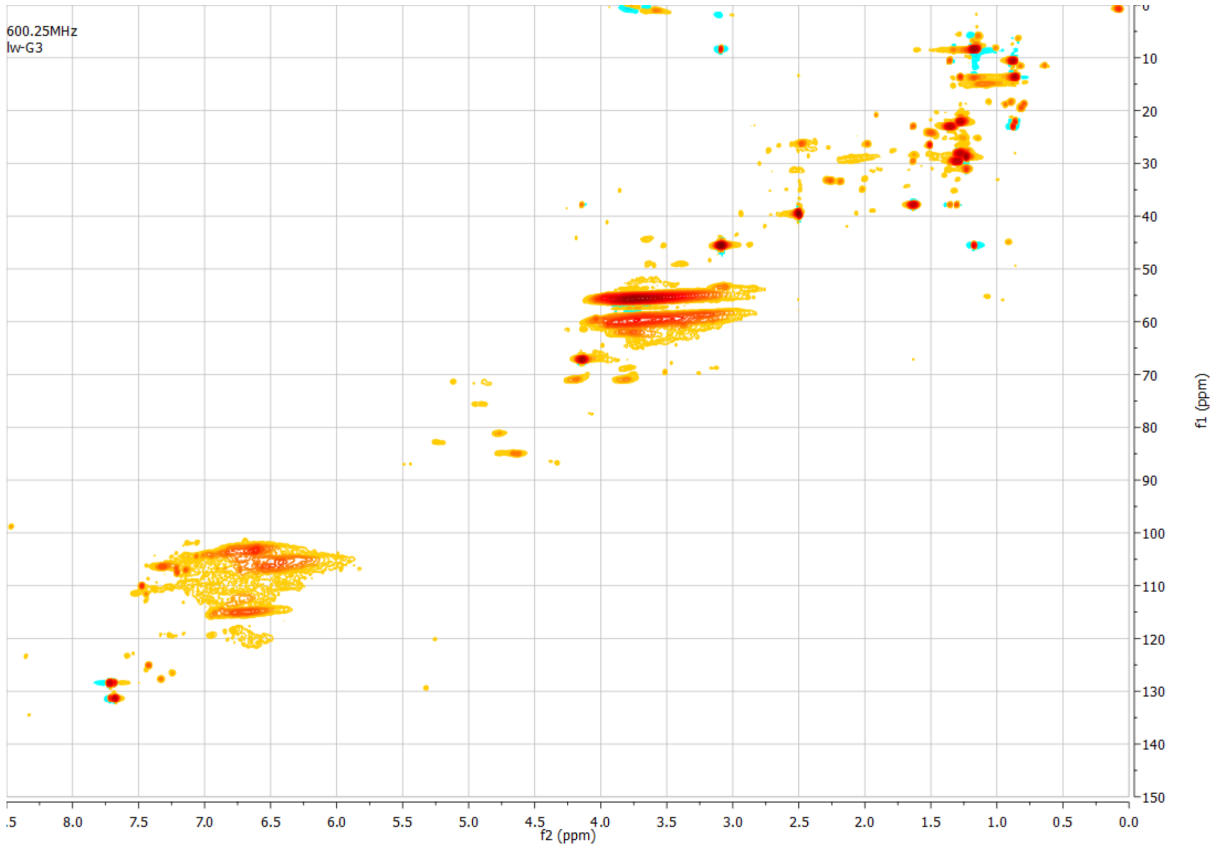

5.8 150°C, 120 min (2 h)

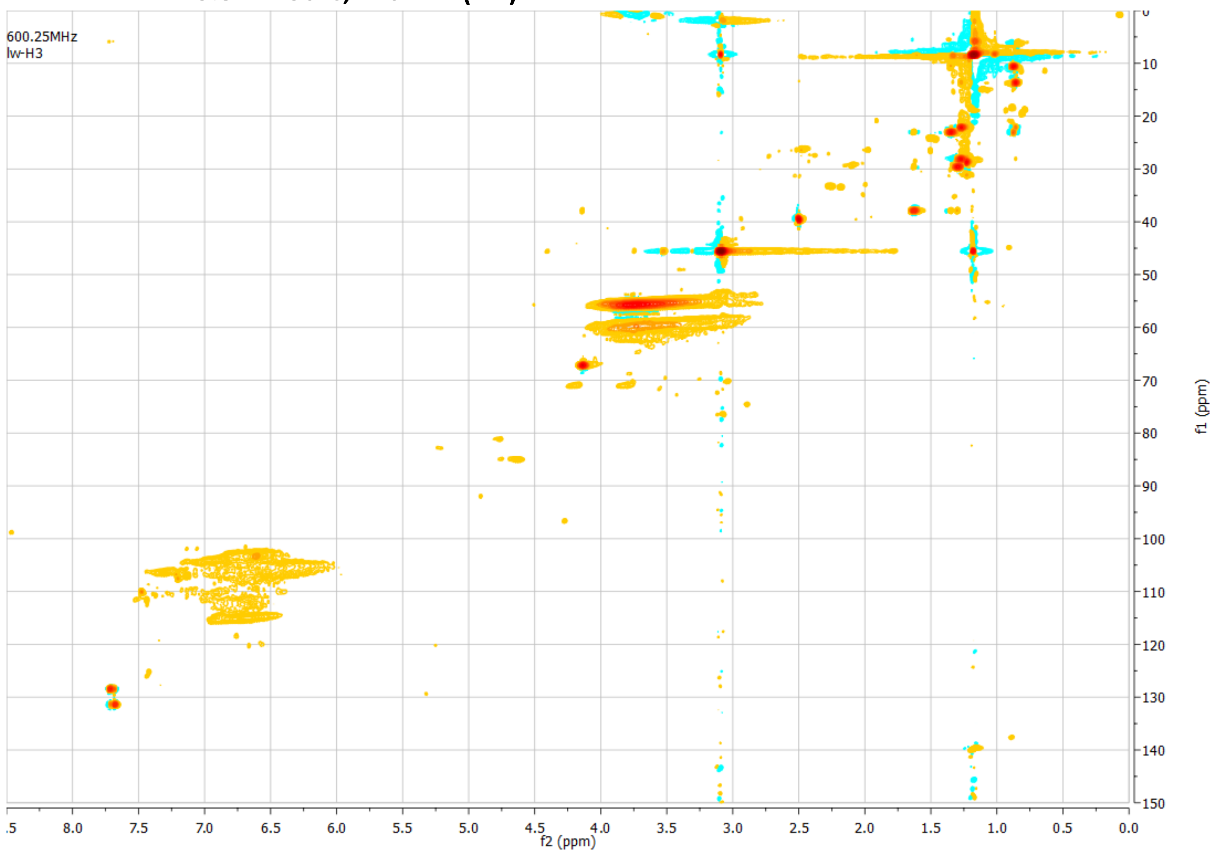

5.9 150°C, 246 min (4.1 h)

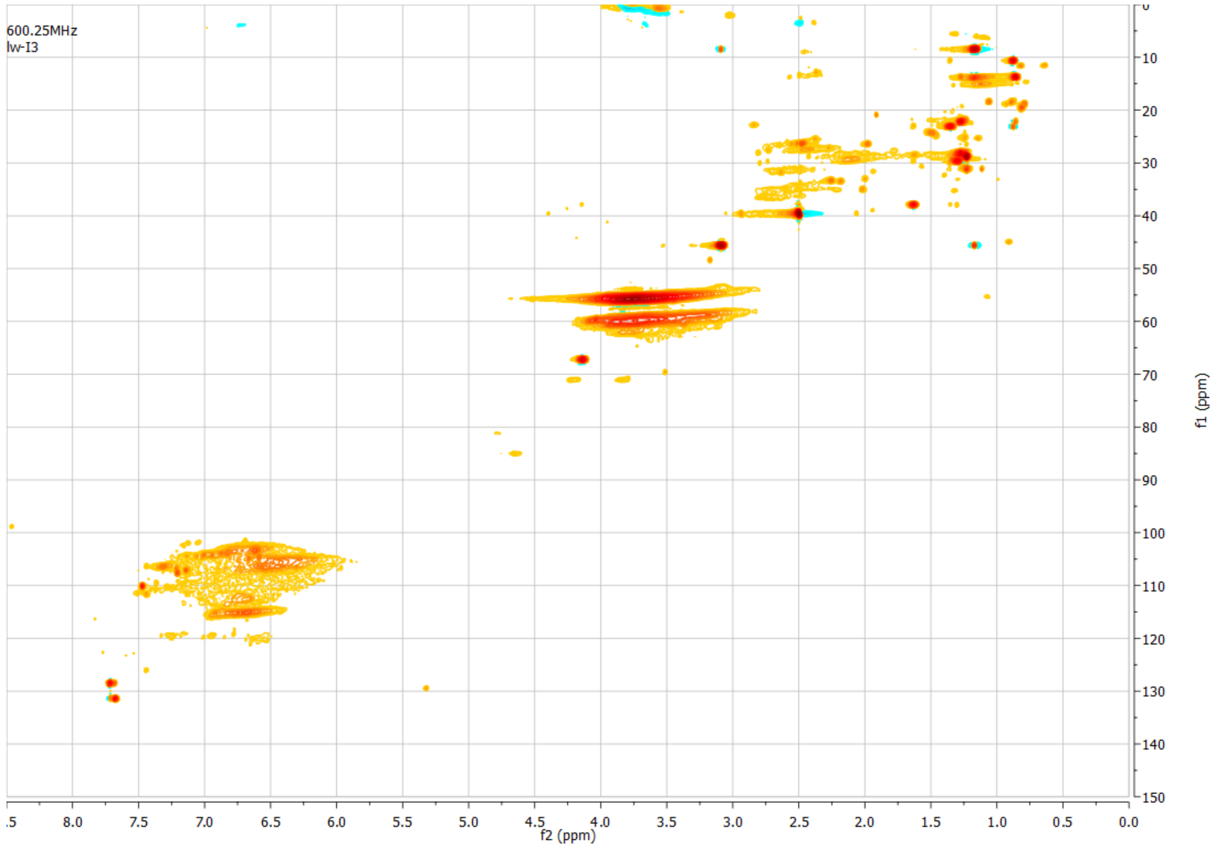

5.10 150°C, 422 min (7 h)

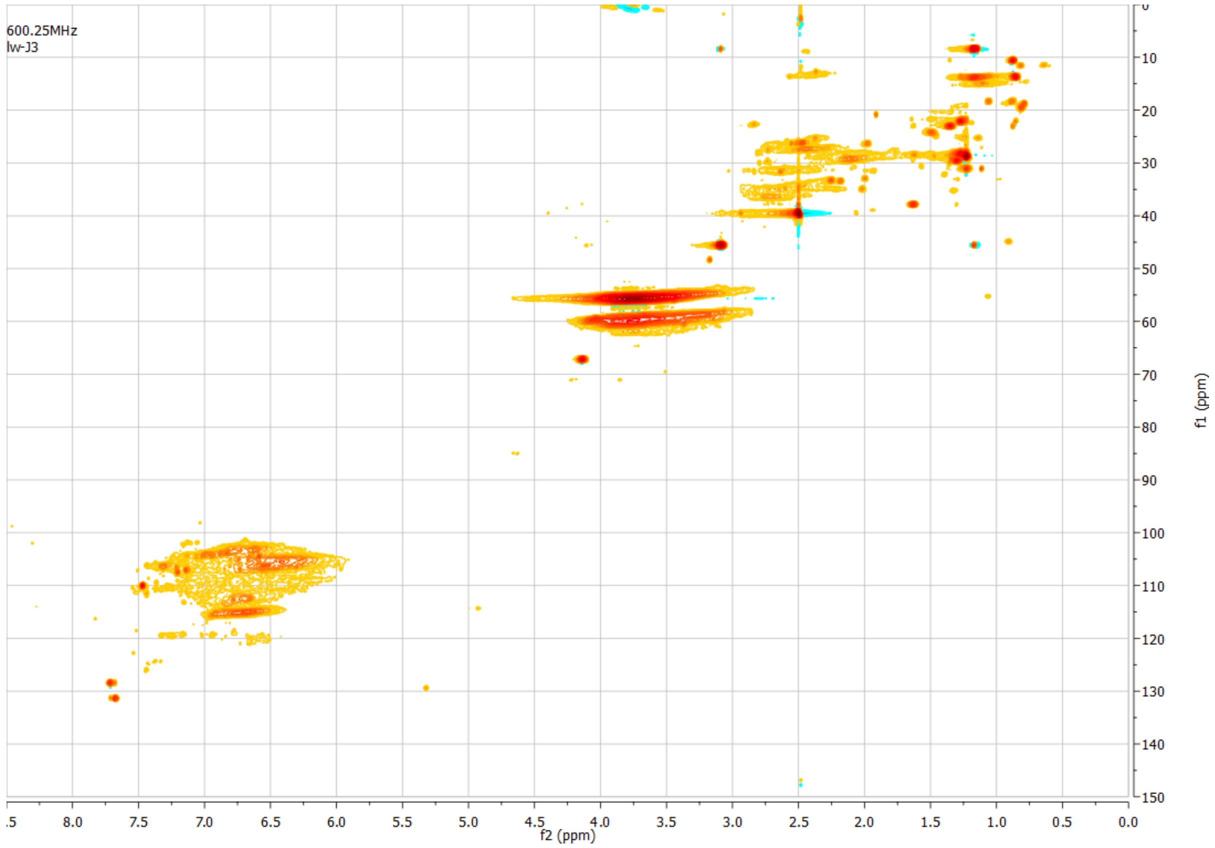

6 Lignin HSQC Spectra - CO_2 Experiments

6.1 H0

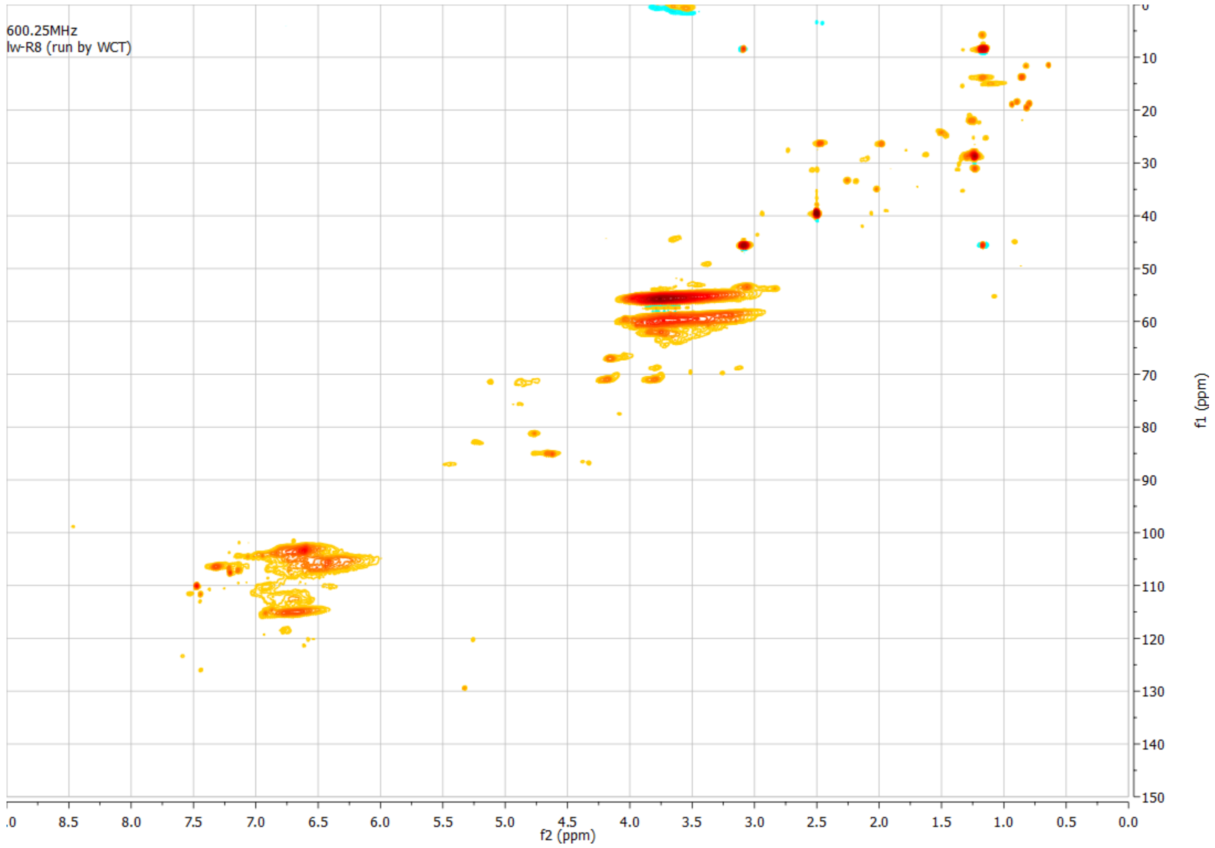

6.2 HL

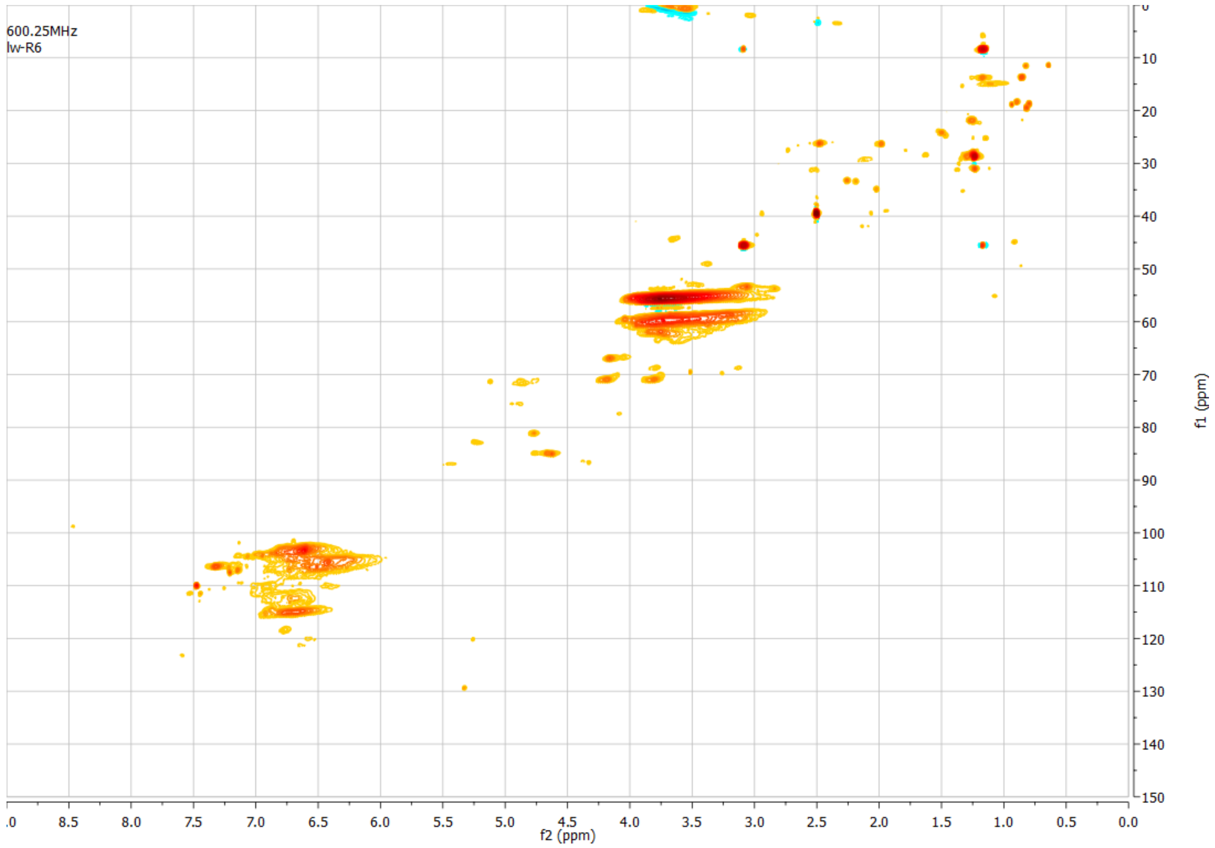

6.3 HH

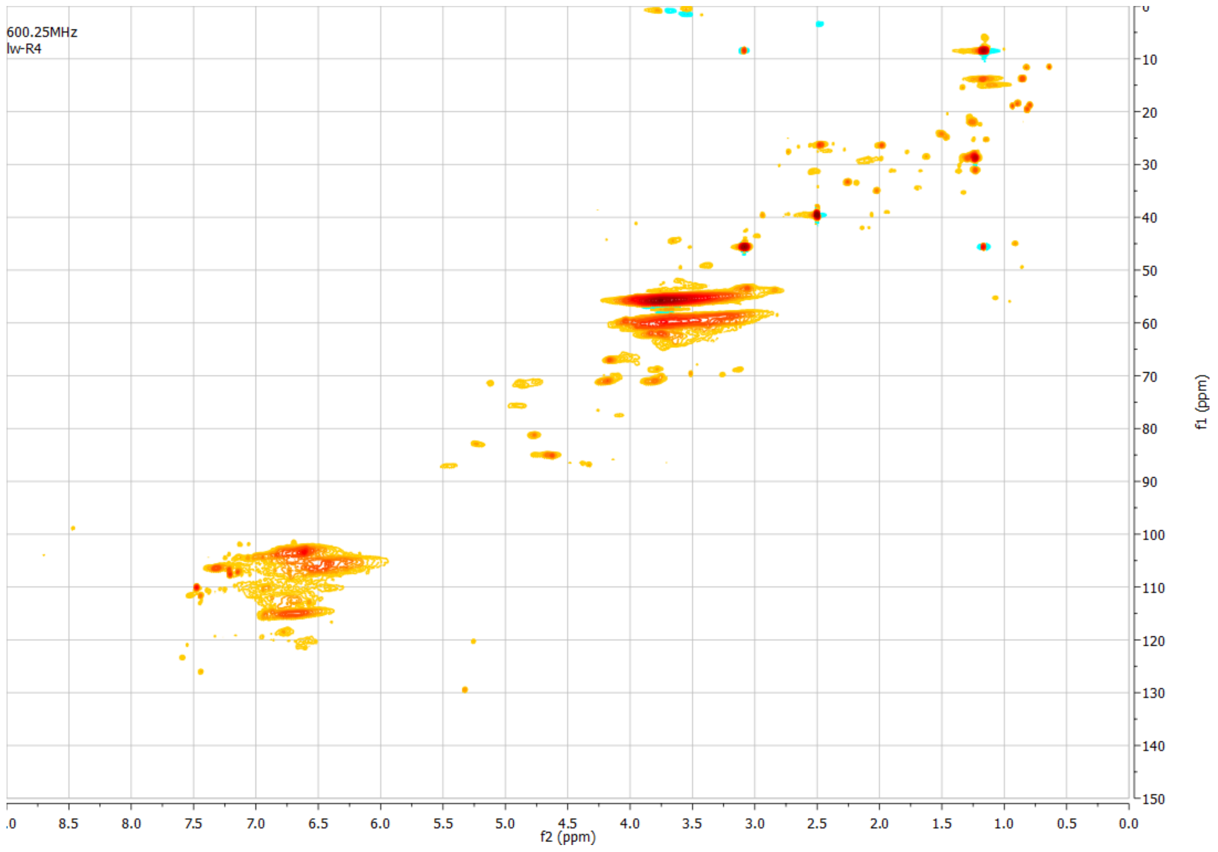

6.4 L0

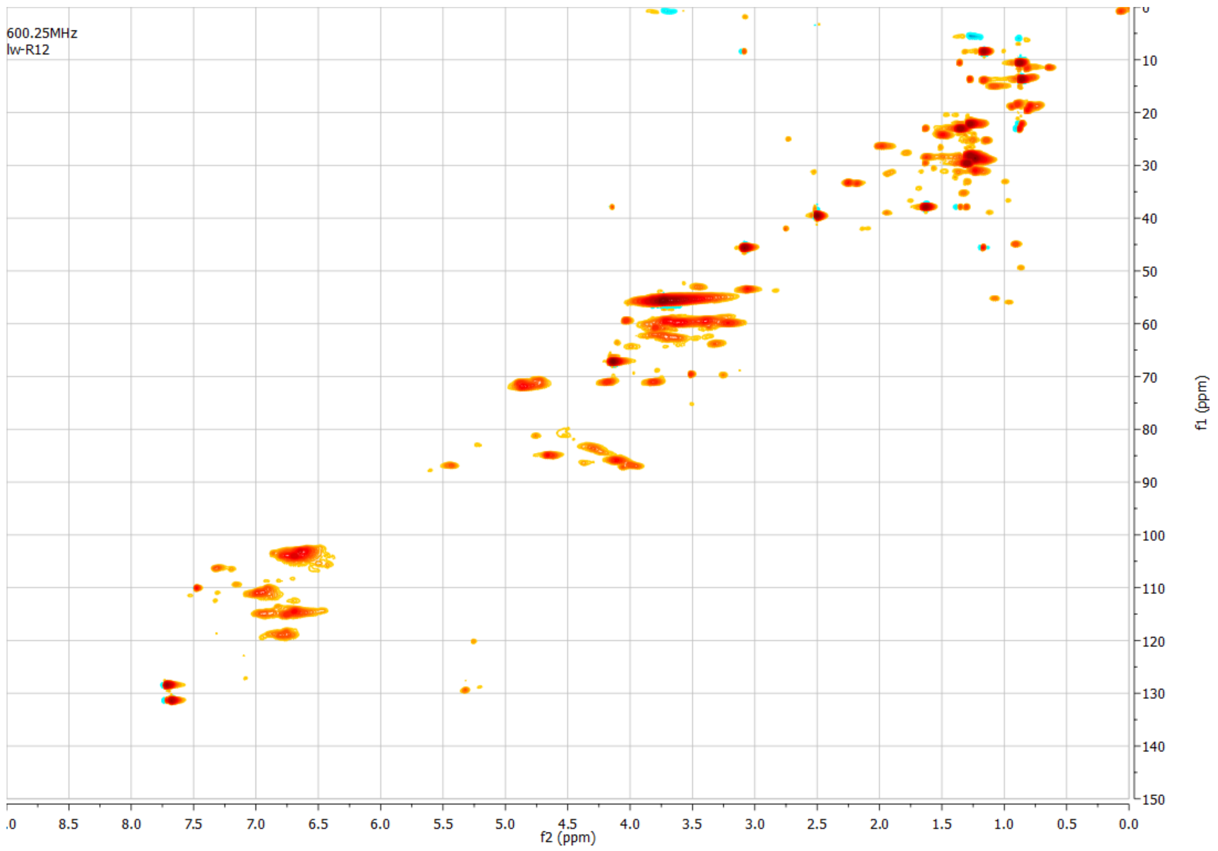

6.5 LL

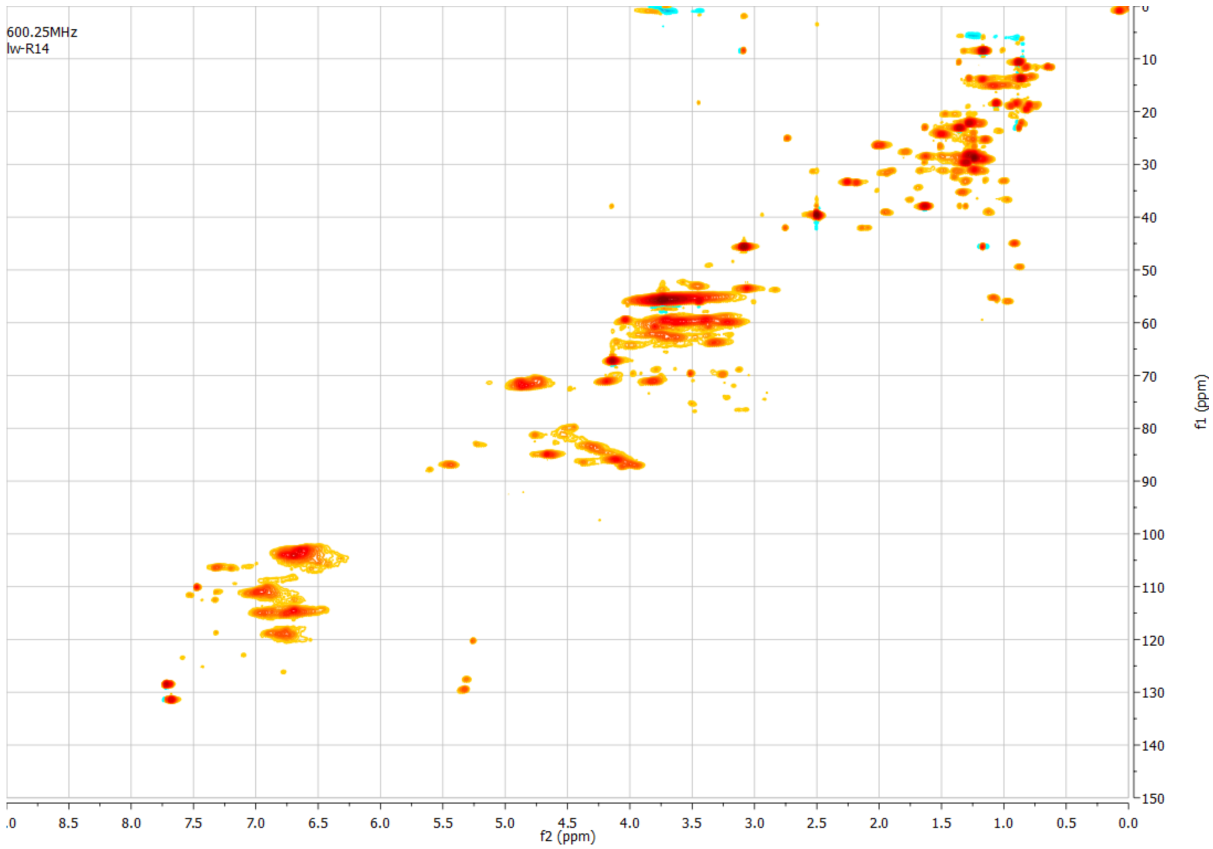

6.6 LH

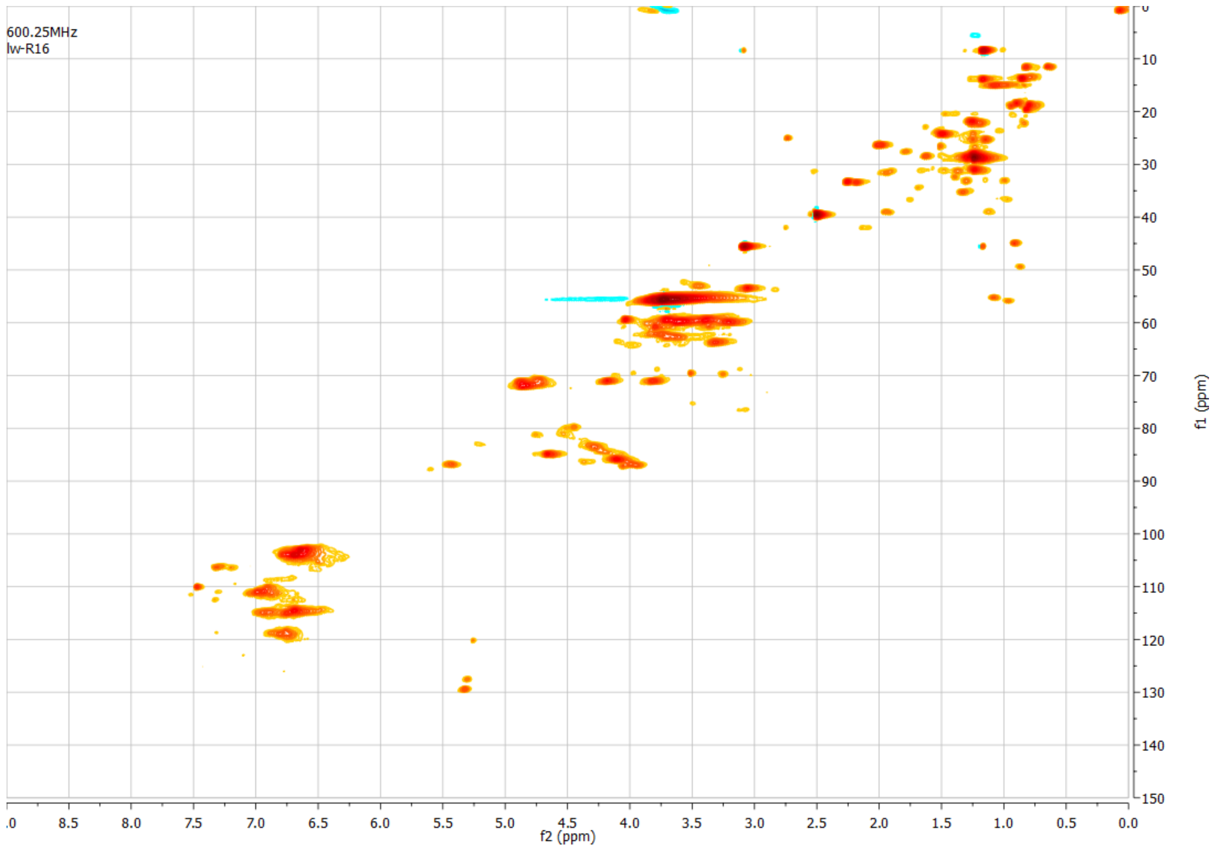

7 References

1. Sluiter, A. *et al.* Determination of Total Solids in Biomass and Total Dissolved Solids in Liquid Process Samples. NREL/TP-510-42621. (2008).
2. Gschwend, F.J. *et al.* Pretreatment of Lignocellulosic Biomass with Low-cost Ionic Liquids. J. Vis. Exp. (114), e54246, doi:10.3791/54246 (2016).
3. Sluiter, A. et al. Determination of Extractives in Biomass. NREL/TP-510-42619. (2008).
4. Sluiter, A. et al. Determination of structural carbohydrates and lignin in Biomass. NREL/TP-510-42618 17. (2012).
5. Resch, M. G. et al. Low Solids Enzymatic Saccharification of Lignocellulosic Biomass. NREL/TP-5100-63351. (2015).
